# Evidence of COVID-19 fatalities in Swedish neighborhoods from a full population study

**DOI:** 10.1038/s41598-024-52988-3

**Published:** 2024-02-06

**Authors:** Sofia Wixe, José Lobo, Charlotta Mellander, Luís M. A. Bettencourt

**Affiliations:** 1 Centre for Entrepreneurship and Spatial Economics, Jönköping International Business School, Jönköping University, Jönköping, Sweden; 2 School of Sustainability, College of Global Futures, Arizona State University, Tempe, AZ USA; 3 Mansueto Institute for Urban Innovation, University of Chicago, Chicago, IL USA; 4 Department of Ecology & Evolution, Department of Sociology, University of Chicago, Chicago, IL USA

**Keywords:** Risk factors, Socioeconomic scenarios

## Abstract

The COVID-19 pandemic has highlighted a debate about whether marginalized communities suffered the disproportionate brunt of the pandemic’s mortality. Empirical studies addressing this question typically suffer from statistical uncertainties and potential biases associated with uneven and incomplete reporting. We use geo-coded micro-level data for the entire population of Sweden to analyze how local neighborhood characteristics affect the likelihood of dying with COVID-19 at individual level, given the individual’s overall risk of death. We control for several individual and regional characteristics to compare the results in specific communities to overall death patterns in Sweden during 2020. When accounting for the probability to die of any cause, we find that individuals residing in socioeconomically disadvantaged neighborhoods were not more likely to die with COVID-19 than individuals residing elsewhere. Importantly, we do find that individuals show a generally higher probability of death in these neighborhoods. Nevertheless, ethnicity is an important explanatory factor for COVID-19 deaths for foreign-born individuals, especially from East Africa, who are more likely to pass away regardless of residential neighborhood.

## Introduction

The ongoing COVID-19 pandemic has reminded us that the features that endow cities with distinctive socioeconomic advantages, such as their vast socioeconomic networks and their density, also make them vulnerable to the spread of pathogens, especially emerging infectious diseases^1–3^. It is by now well known in the social sciences that the effects of urban environments on individual-level outcomes are mediated, to a large extent, by local residential contexts, i.e., neighborhoods^4–11^. There were concerns from the very start of the COVID-19 pandemic that the manner and pace of contagion and the impacts of the disease would be greatly magnified by neighborhood characteristics, with neighborhood-level inequalities expressed as concentrated poverty, segregation, and deprivation amplifying the virus’s infectivity and deadly consequences. These expectations support a set of hypotheses that we test here in the context of Sweden, using the country’s full population records of mortality. The aim of the paper is thus to analyze whether individuals residing in ethnically segregated and socioeconomically weak neighborhoods were more likely to die with COVID-19. We specifically contribute to existing literature by conditioning COVID-19 deaths on overall rates of mortality. Given our access to geocoded individual micro-level data, we are thus able to trace out whether individuals in disadvantaged neighborhoods had a higher-than-expected risk of dying due to the pandemic.

Expectations for neighborhood effects resulting in disproportionate impacts of the COVID-19 pandemic on disadvantaged local communities follow from decades of observational and empirical work in sociology, economics, and public health sciences^12,13^. This literature gained a renewed focus since the 1980s with the work of Wilson, who characterized processes of local and cumulative disadvantage in deindustrializing US cities, which created mass unemployment among working class Black communities and facilitated the formation of inner-city marginalized neighborhoods^14^. Analyses of neighborhood effects have grown substantially in recent years supporting a multidisciplinary account of the origins and consequences of spatially concentrated local disadvantage^15–18^. More recent empirical work, using detailed data sources, such as tax records, has better established the critical importance of temporal exposure to disadvantaged neighborhoods, especially during childhood, with life course consequences affecting future income, family structure, health, and life expectancy^19–21^.

The empirical richness and methodological diversity which characterize the investigation of neighborhood effects studies suggest complicated chains of causality underlying local disadvantage involving interlocked social, economic, cognitive, and behavioral phenomena. Neighborhood effects are clearly identifiable through spatially resolved maps of socioeconomic outcomes in any city, which often manifest variations within short distances of about a kilometer^5^. However, delineating neighborhoods remains problematic, as often different definitions are used. Unlike other geographies for which socioeconomic data are typically collected by government agencies, neighborhood-level data are usually not collected^22^. The ubiquity and pervasiveness of neighborhood effects, also makes them difficult to parse out due to the presence of confounding factors operating at different spatial and social scales^15,23,24^.

The transmission and health consequences of an emerging infectious disease like COVID-19 was expected to be greatly modulated by neighborhoods’ spatially embedded characteristics, creating strong place-based inequalities in outcomes. There are aspects of neighborhoods, like social cohesion and connectedness to different communities, that extend beyond its physical boundaries, and which can play a role in determining access to prevention and treatment resources. But with regards to transmission of pathogens and access to medical care, early concerns on how the COVID-19 pandemic would be experienced by different local populations have indeed been borne out by numerous recent studies on urban areas and neighborhoods throughout the globe. The evidence indicates that residents of disadvantaged neighborhoods experienced a greater epidemic burden^25–31^. Among these findings is the role of neighborhood poverty at increasing the likelihood of becoming infected, incurring more severe cases, and the probability of dying once infected^32–34^ also in Sweden^35,36^.

A critical question is how to distinguish between the effects of individual-level characteristics and spatial sorting versus the “ecological” (collective) characteristics of neighborhoods. Furthermore, determining the exact cause of death, especially in the context of COVID-19, can be challenging for several reasons. This challenge of attribution arises when establishing statistical associations with morbidity and mortality from any cause, in the sense of whether, for example, a death is associated to one’s individual socioeconomic status, medical background, or neighborhood effects^37–39^. People often have multiple health conditions (comorbidities) that interact with each other. COVID-19 may exacerbate existing health issues, making it difficult to attribute a death solely to the virus. Not everyone who dies with COVID-19 symptoms is tested for the virus. Conversely, some deaths attributed to COVID-19 might not have been directly caused by the virus. This variability affects accurate reporting. Attributing deaths to COVID-19 based on symptoms alone may lead to overreporting.

In the case of COVID-19 mortality, a variety of confounding individual factors have emerged from empirical studies and clinical observation, including diet, obesity, pre-existing medical conditions, mental health challenges, environmental pollution, and maybe most importantly, age. Several studies also point to differences in COVID-19 mortality across ethnic groups, with ethnic minorities being more severely affected during the pandemic^40–42^. All these factors are likely to have detrimental effects on the seriousness of infection and increasing risks of mortality, thus clouding our ability to assess specific place-based neighborhood effects^43–47^.

We availed ourselves of Swedish micro-level data sources from 2020 covering the full population aged 15 and above, and combined them in a novel way, so as to investigate how individuals’ socioeconomic, residential and health characteristics affected their likelihood of dying with COVID-19, given that they pass away at all. While it is hard to truly disentangle any specific effect, we think the resulting data construct provides a nearly unique perspective with which to investigate the presence and strength of neighborhood effects in relation to COVID-19 fatalities. We argue that to fully understand, and to not overestimate, the impact of neighborhoods on COVID-19 fatalities, it is necessary to account for spatial variations in overall probabilities to die. Indeed, we find that individuals in ethnically segregated, and socioeconomically weak neighborhoods are more likely to die. Furthermore, we find that they are generally *not more likely* to die with COVID-19 given the already higher expected risk of dying. A relatively common approach in the literature on COVID-19 is also to analyze excessive deaths across, for example, regions and population groups^48–50^. Although presenting interesting findings, these studies are conducted at an aggregate—and not individual—level and typically do not attempt to explain *why* certain individuals are more prone to die with COVID-19. As such, our study provides new evidence on neighborhood effects on individual health outcomes in the context of a world-wide pandemic, concluding that health deterioration in disadvantaged neighborhoods is a severe issue also in non-pandemic times.

## Material and methods

What is needed to clearly identify and quantify neighborhood effects on COVID-19 severity and mortality are data with sufficient individual-level and spatial resolution so that analyses of greater sophistication than generalized (linear) correlations can be performed. Further, there is a need for a quantitative comparison standard—in this case, mortality due to other causes—to determine if and how COVID-19 specific mortality significantly differs. Also needed is an identification of “neighborhood” that is not an ad hoc use of another type of spatial unit constructed for purposes other than the examination of neighborhood effects.

### Description of the datasets

This analysis builds on several register data sources. The study does not involve direct human participants but is fully based on anonymized register data. It was approved by the Swedish Ethical Review Authority with diary numbers 2018/174-31 and 2020-05,497 and carried out in accordance with relevant guidelines and regulations. We obtained comprehensive geo-coded micro-level data on the total population of Sweden aged 15 or above. This dataset consists of 8,477,638 individual observations, from Statistics Sweden (the governmental agency responsible for providing official socioeconomic statistics for the country) for the year 2019. These data are collated from the Swedish national register known as the “Longitudinal Integrated Database for Health-Insurance and Labor-Market Studies” (LISA). It provides detailed quantitative indicators for every individual, family, and workplace, as well as information about place of residence down to the neighborhood level. Data in this register are in turn assembled by various Swedish official authorities, such as the Income and Tax Register and the Social Insurance Agency. The database is updated on a yearly basis and applies to a whole year as the temporal measurement period, using the population on the 31st of December. The variables in the database are grouped by demographic characteristics (age, gender), education and training attainment, employment status, income and to what extent an individual has been financially compensated via the social insurance system, family characteristics (e.g., civil status, number of children in the household), and workplace establishment and firm information (e.g., industry, number of employees, occupational structures). Information is also available about individuals’ residential housing arrangement such as the number of rooms in their dwelling. A detailed description of the variables utilized in this study is available in the supplementary material (Tables S1-S3).

**Figure 1:**
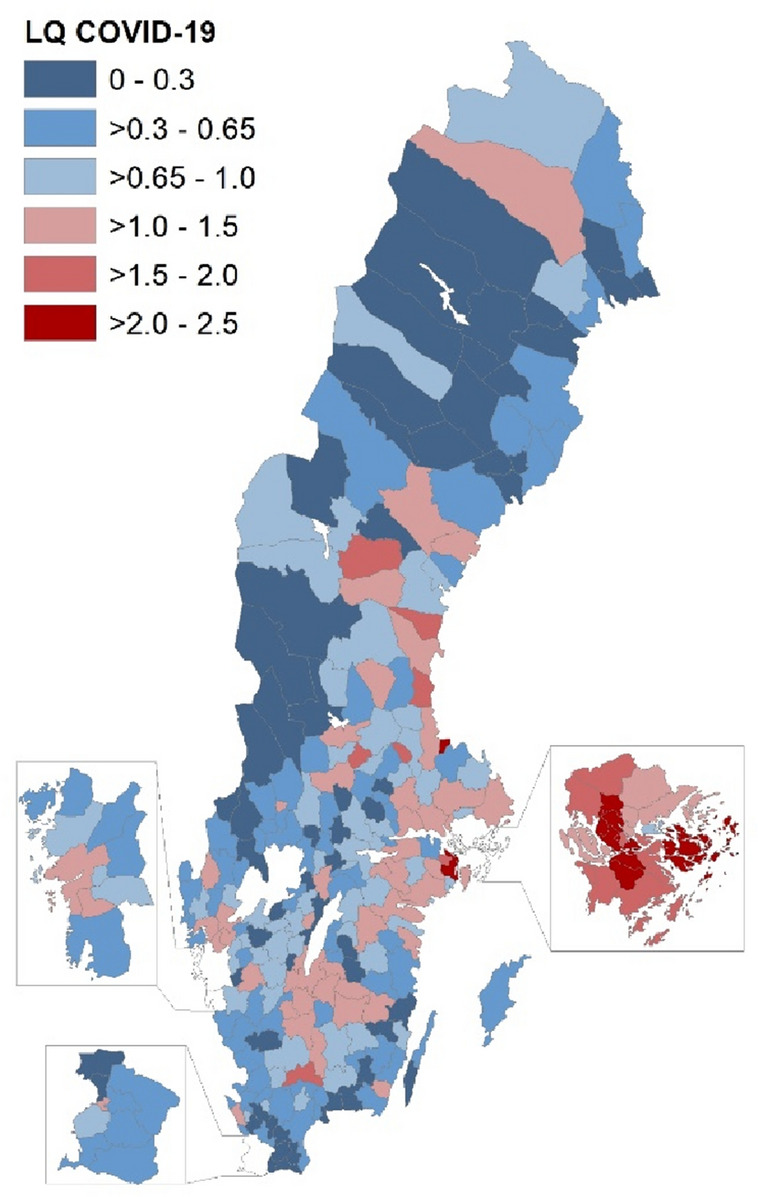
Relative COVID-19 deaths per municipality 2020 ($LQ=\frac{COVID-19 \,deaths\, in\, municipality/COVID-19 \,deaths \,in \,country}{All \,deaths \,in \,municipality/All \,deaths \,in \,country}$).

To the already existing full-population data set, we have linked additional data from the “Cause of Death Register” constructed by the Swedish National Board of Health and Welfare, covering all deaths in the country during 2020. This dataset includes information on causes of death (including multiple causes), date of death as well as information on diabetes diagnosis. Diabetes is important to control for as it is highlighted as a risk factor for COVID-19 deaths. Since individuals with underlying diseases face a higher risk of severe infection and death in COVID-19 *and* are more likely to reside in disadvantaged areas ^51,52^, we control for multiple causes of death to not overestimate the impact of neighborhood characteristics. In total, 97,374 individuals passed away in Sweden during 2020, of which 97,197 can be matched to the 2019 micro-data. We matched against the year 2019 since the individual characteristics register data from Statistics Sweden is based on the last day of the year and thus the individuals who died would not be registered during the year 2020. Of those, 10,168 individuals had COVID-19 as a cause of death, 4.8 percent had COVID-19 as the sole cause of death, 90 percent of the dead with COVID-19 during 2020 were aged 70 and above, and 53 percent were male. To get a first glimpse of the spatial variation of individuals dying with COVID-19 in Sweden, Fig. 1 shows the distribution of COVID-19 deaths across municipalities, relative to the overall number of deaths in each municipality. COVID-19 cases are mainly overrepresented in the Stockholm region, as shown by the darker red colors in this area.

The European Union statistical agency reported that Sweden had an excess mortality rate of 7.7 percent in 2020 in a comparison with the previous 4 years^53^. Of the 30 countries for which data are available, 21 had a higher excess mortality rate than Sweden. However, Sweden had significantly higher excess mortality than its Nordic neighbors Denmark (1.5 per cent) and Finland (1.0 per cent).

In the estimations of COVID-19 deaths, we control for potential virus exposure in each municipality by counting the number of COVID-19-infected in the municipality and region (weighted by distance to the municipality in question) in the week preceding the individual’s death. As COVID-19 hit different municipalities at different times during the year, this is a more relevant measure than simply controlling for time (for example, weeks or months) in general.

Data processing and all statistical analysis, including the generation of Figs. 4, 5 and 6 are performed using STATA, version 18^54^. Maps are created using ArcGIS software by Esri, version 10.8.1^55^.

### Spatial units of analysis

To analyze how the neighborhood of residence affected the likelihood of dying with COVID-19, we take advantage of the geographic information in the data from Statistics Sweden to construct neighborhood characteristics based on ethnic background, education level, and incomes. There is no unique neighborhood definition in Sweden, but several definitions have been developed during different time periods. The *SAMS* definition (Small Areas for Market Statistics) was introduced in 1994 and has often been used in Swedish studies of neighborhood effects^56^. However, this definition has been criticized because these neighborhood areas are not as homogeneous as it is sometimes assumed. In 2018, SAMS was replaced by a new neighborhood definition known as *DeSo* (Demographic Statistical Areas) that took into account changes in buildings and infrastructure, since SAMS was developed, which means that the SAMS definition was no longer considered to meet the existing needs. We define neighborhoods according to Statistics Sweden’s recent (2020) classification of *Regional Statistical Areas* (*RegSO*), which builds on the DeSo definition and delineates 3,363 units of analysis covering the whole nation. This geographic specification is especially advantageous for studying neighborhood effects since the purpose of RegSO is to allow for statistical studies that assess socioeconomic segregation^67^. The final RegSo classification was introduced in 2020 and is intended to remain unchanged over time. A benefit of this classification of neighborhoods is that location-specific demographic conditions are defined with spatial (neighborhood) borders following, e.g., streets, waterways, and railways. RegSO areas thus represent actual neighborhoods rather than arbitrary administrative constructs.

**Figure 2:**
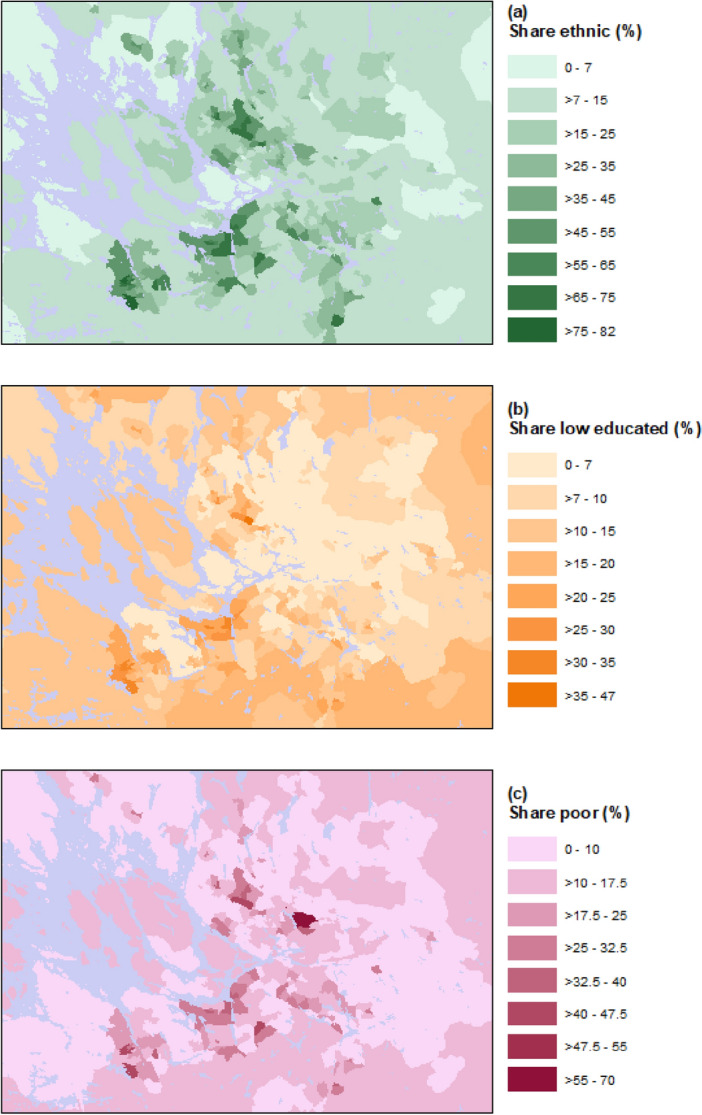
Share of foreign-born (panel **a**), low educated (panel **b**), and in risk of poverty (panel **c**) per neighborhood in Stockholm and surrounding areas (blue color indicates water).

**Figure 3:**
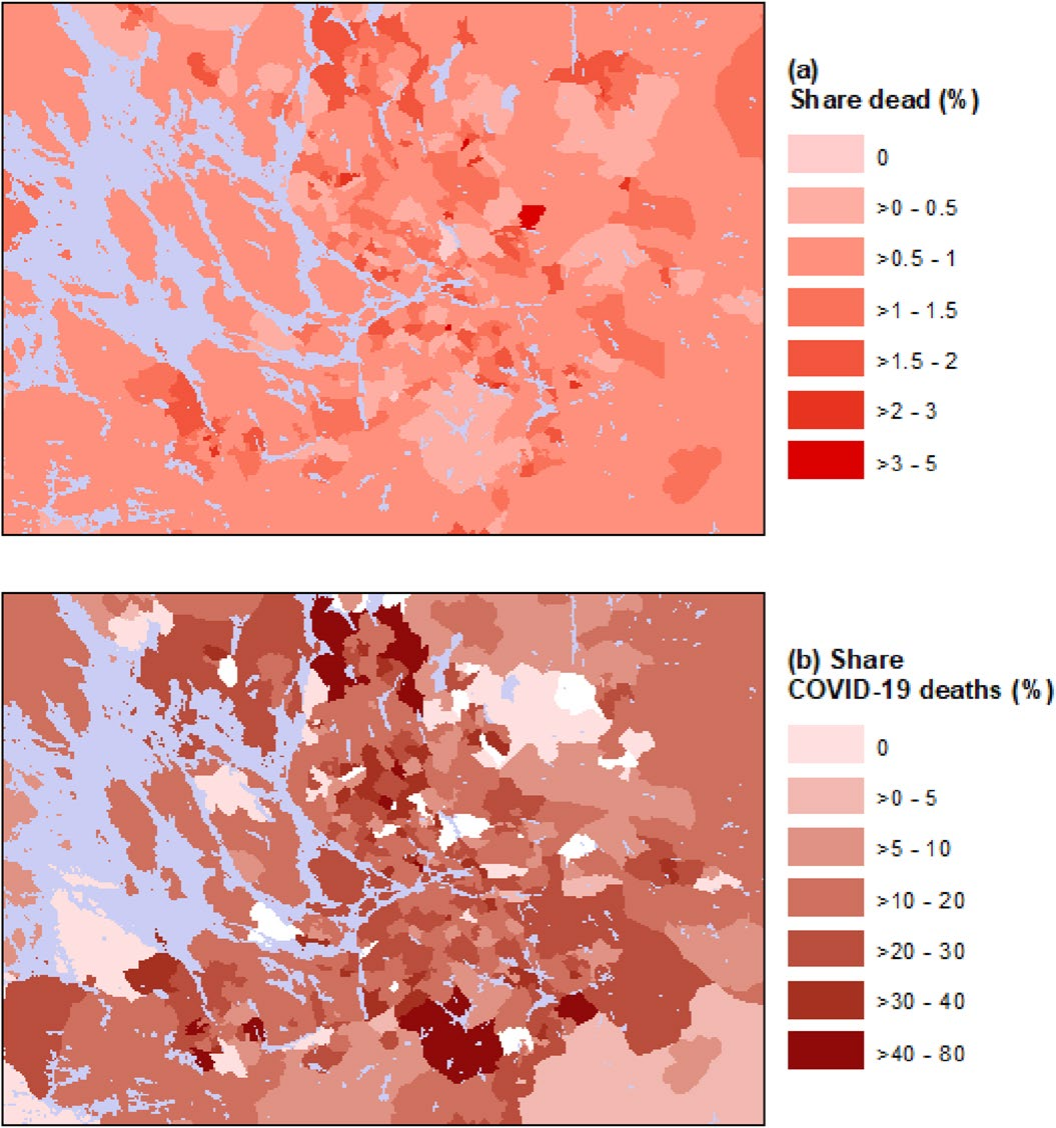
Share of dead (dead relative to total population) (panel **a**) and share of COVID-19 deaths (COVID-19 deaths relative to total deaths) (panel **b**) per neighborhood in Stockholm and surrounding areas (blue color indicates water). Panel b does not include data for neighborhoods with fewer than five dead in total.

Based on the residential geo-coded information, we can construct the socio-economic characteristics of the neighborhood in which an individual resides. We measure neighborhood characteristics in terms of the share of residents in the neighborhood who (1) were born in a non-Nordic/EU15 country (population aged 15 +), (2) had only an elementary school degree as highest educational attainment (population aged 20–64, thus excluding individuals in school age and the older generation who were overall less likely to attend high school and higher education levels), and (3) are at the risk of poverty, defined as having a disposable income below 60 percent of the corresponding national median (population aged 20 + , thus excluding individuals in school age)^57^. Figure 2 illustrates the neighborhood characteristics focusing on the areas in and around Stockholm. Figure 3 shows the overall deaths and COVID-19 deaths for the same geographic area. Further descriptive statistics for the neighborhood variables are available in the supplementary material (Fig. S1-S7, Table S4).

### Estimation of the probability of dying with COVID-19

Differences in individuals’ propensity to die with COVID-19 across neighborhoods are hypothesized to be caused by different socio-economic conditions. At the same time, recent studies point to that also general death rates vary across neighborhoods, resulting in lower life expectancies in disadvantaged neighborhoods^58,59^. There may thus be an issue of selection bias when estimating neighborhood effects on the likelihood of dying with COVID-19 coming from the fact that individuals residing in certain types of neighborhoods are more likely to die overall. Hence, we estimate neighborhood effects on the probability of dying with COVID-19 *conditional* on the probability of dying in general, using the Heckman selection model^60–62^. Even though this may not be the typical selection bias problem identified in previous literature, the Heckman two-step approach is a feasible and general-enough method to deal with selection bias. Originally proposed to correct for non-randomly selected data samples, the technique explicitly models the selection probability of each observation (the selection equation) together with the conditional expectation of the dependent variable (the outcome equation).

That selection bias does indeed exist in our case is supported by the estimations showing a rho, *ρ*, significantly different from zero and a rejection of the hypothesis of independent equations (using the Wald test). Additionally, running probit estimations on COVID-19 deaths without selection bias correction produce vastly different estimates of several variables (Table S6). Of particular importance for this paper is that ordinary probit estimations tend to overestimate the importance of neighborhood effects on COVID-19 deaths, confirming the need for the two-step procedure.

In the first step of the Heckman selection model, the probability of dying for a population on a given year is estimated. The canonical specification for this relationship is a probit regression of the form:

1 $${s}^{*}=Z\gamma +{u}_{1},$$

where $s$^*^ is an unobserved latent variable, $Z$ is a vector of explanatory variables displayed in Table S1, $\gamma$ is a vector of unknown parameters (regressors), and ${u}_{1}$ is standard normally distributed error term. The binary outcome variable, $s=\mathrm{0,1}$(meaning death or no death over the time period), are observed if

2 $$s=\left\{\begin{array}{c}1 {\text{ i}}{\text{f}} {\text{ s}^{*}}>0\\ 0 {\text{ i}}{\text{f}}{\text{ s}^{*}}\le 0\end{array}\right.$$

The probability of being selected (that is, dying) is specified by

3 $${\text{Prob}}\left(s=1|Z\right)={\text{Prob}}\left({s}^{*}>0\right)={\text{Prob}}\left({u}_{1}<Z\gamma \right)=\Phi \left(Z\gamma \right)$$

where $\Phi$ is the cumulative distribution function of the standard normal distribution. The model estimation, in terms of the coefficients $\gamma$, yields results that can be used to predict the death probability for each individual.

In the second step of estimation, we correct for “self-selection” (the fact that COVID-19 deaths are only observed in “the population of dead”) by incorporating a transformation of these predicted individual probabilities as an additional explanatory^61^. Analogously to the first step, the probability of dying with COVID-19 may be specified using another latent variable

4 $${y}^{*}=X\beta +{u}_{2}$$

where $X$ is a vector of explanatory variables (Table S1) and where we can observe the binary outcome $y=\mathrm{1,0}$ (specific death with COVID-19), given that an individual has died such that

5 $$y=\left\{\begin{array}{c}1 {\text{ i}}{\text{f}} {\text{ y}}^{*}>0\\ 0 {\text{ i}}{\text{f}} {\text{ y}^{*}}\le 0\end{array}\right.$$

The assumptions of the error terms are ${u}_{1}\sim N\left(\mathrm{0,1}\right)$, ${u}_{2}\sim N(\mathrm{0,1})$ and ${\text{corr}}\left({u}_{1},{u}_{2}\right)=\rho \ne 0.$

Thus, we get the conditional expectation of dying with COVID-19 according to

6 $$E\left[y|X, s=1\right]=X\beta +E\left[{u}_{2}|X, s=1\right]$$

Under the error terms assumptions, we have that

7 $$E\left[y|X, s=1\right]=X\beta +\rho \lambda \left(Z\gamma \right)=X\beta +\rho \frac{\phi (Z\gamma )}{\Phi (Z\gamma )}$$

where ρ is the correlation between unobserved determinants of the probability of dying ${u}_{1},$ and unobserved determinants of the probability of dying with COVID-19, ${u}_{2}$, and $\lambda$ is the inverse Mills ratio, evaluated at $Z\gamma$. In other words, sample selection is viewed as a form of omitted-variable bias, as conditional on both $X$ and on λ as if the sample is randomly selected. (As usual, $\phi$ denotes the standard normal density function.)

We estimate the Heckman selection model on the total population of Sweden aged 15 or above as well as the population aged 70 and above since the latter comprise the bulk of COVID-19 deaths. Additionally, we perform separate estimations for different regional types, by partly identifying municipalities belonging to the Stockholm region and partly distinguishing between metropolitan, urban, and rural municipalities following the classification by the Swedish Agency for Economic and Regional Growth^63^.

## Results

### Neighborhoods and COVID-19 mortality

We estimated the probability of dying with COVID-19 conditional on the probability of dying in general using a two-step Heckman Probit regression model^60–62^. In the estimation, we examine to what extent neighborhood characteristics, in terms of ethnicity, education level, and income level, significantly explain the probability of dying with COVID-19 (step 2) conditional the probability of dying in general (step 1), while still controlling for other individual and regional characteristics. The following results Tables 1, 2, 3, 4, 5 and 6 are organized based on neighborhood characteristics, in terms of the share of residents in the neighborhood who are (1) foreign born, (2) low educated, and (3) at the risk of poverty. All coefficients in the tables are expressed as average marginal effects (AMEs), in other words the average change in probability of dying (step 1) and dying with COVID-19 (step 2) when the explanatory variable in question changes by one unit. For categorical variables, the AMEs show the change in probability in relation to the base category.

Table 1 presents the estimation results of various socioeconomic characteristics on the general likelihood of death (all causes), controlling for individual and regional characteristics.

**Table 1:** Marginal effects for the probability of dying at individual level (selection equations).

	(1a)		(2a)		(3a)	
	i) Ethnic		ii) Low education		iii) Poverty	
Neighborhood characteristics						
	> 7–15%	− .0001	> 7–10%	.0003*	> 10–17.5%	.0002
	> 15–25%	.0004*	> 10–15%	.0007*	> 17.5–25%	.0007*
	> 25–35%	.0009*	> 15–20%	.0014*	> 25–32.5%	.0013*
	> 35–45%	.0018*	> 20–25%	.0019*	> 32.5–40%	.0020*
	> 45–55%	.0021*	> 25–30%	.0019*	> 40–47.5%	.0024*
	> 55–65%	.0028*	> 30–35%	.0032*	> 47.5–55%	.0026*
	> 65–75%	.0029*	> 35%	.0043*	> 55%	.0011
	> 75%	.0052*				
No-go-zone	−.0004		−.0006*		−.0010*	
Regional characteristics						
Population density	− .0001*		− .0001		− .0001	
Regional type						
Urban	− .0003*		− .0000		− .0002	
Metro	.0003		.0009*		0.0006*	
Individual characteristics						
Ethnic background						
Nordic	.0008*		.0007*		.0008*	
EU15	− .0010*		− .0010*		− .0010*	
West Balkan	− .0003		− .0001		− .0001	
East Europe	− .0015*		− .0014*		− .0014*	
Middle East	− .0020*		− .0018*		− .0017*	
East Africa	− .0026*		− .0026*		− .0024*	
North–South-West Africa	− .0033*		− .0032*		− .0032*	
South-Central Asia	− .0032*		− .0031*		− .0030*	
Southeast-East Asia	− .0028*		− .0027*		− .0027*	
South-Central America	− .0032*		− .0031*		− .0030*	
North America-Oceania	− .0026*		− .0023*		− .0024*	
Education						
High school	− .0016*		− .0016*		− .0016*	
Shorter higher education	− .0033*		− .0032*		− .0033*	
Longer higher education	− .0041*		− .0040*		− .0042*	
Income	− .0006*		− .0006*		− .0006*	
Age						
30–39	.0003*		.0003*		.0003*	
40–49	.0011*		.0011*		.0011*	
50–59	.0029*		.0029*		.0029*	
60–69	.0082*		.0082*		.0082*	
70–79	.0211*		.0211*		.0211*	
80–89	.0596*		.0596*		.0596*	
90 +	.1415*		.1413*		.1413*	
Female	− .0047*		− .0047*		− .0047*	
Civil status						
Single with child	− .0040*		− .0040*		− .0040*	
Married	− .0031*		− .0031*		− .0031*	
Married with child	− .0057*		− .0057*		− .0057*	
Frontline worker	− .0018*		− .0018*		− .0018*	
Elderly home	.0280*		.0281*		.0281*	
House type						
Tenant-owned (apartment)	− .0020*		− .0019*		− .0020*	
Owner-occupied (house)	− .0027*		− .0027*		− .0027*	
Observations	8,477,638		8,477,638		8,477,638	
Wald Chi2	44,901*		45,447*		45,037*	

*Notes:* Dependent variable = 1 if dead, zero otherwise. * denote significance at 1% level.

The results on the neighborhood variables are in line with the expectations, showing that individuals residing in more ethnically segregated and socioeconomically weaker neighborhoods have a greater probability of dying. Starting with ethnicity, neighborhoods with higher shares of foreign born (> 75%) have a marginal effect of 0.0052, which is about double the marginal effect of all other ethnic share categories. The marginal effect on general death rates is insignificant for neighborhoods with the lowest shares of foreign born but increases to 0.0004 and 0.0009 for neighborhoods with 15–35 percent foreign born, increasing further with higher shares of foreign born. We find similar patterns for the influence of education, where neighborhoods with higher shares of low-educated individuals display a higher probability of death from all causes. The marginal effect on mortality for the group with the highest shares of low educated is 0.0043, compared to 0.0003 and 0.0007 for the groups with the lowest (better educated). For poverty, the patterns are similar: The higher the share of individuals in poverty, the higher the marginal effects on the general probability of dying. However, for the category with the highest shares of the poor (> 55%), we observe a decline in the death probability because this category features low-income student areas in college towns (this is discussed further below in regards to COVID-19 fatalities).

**Figure 4:**
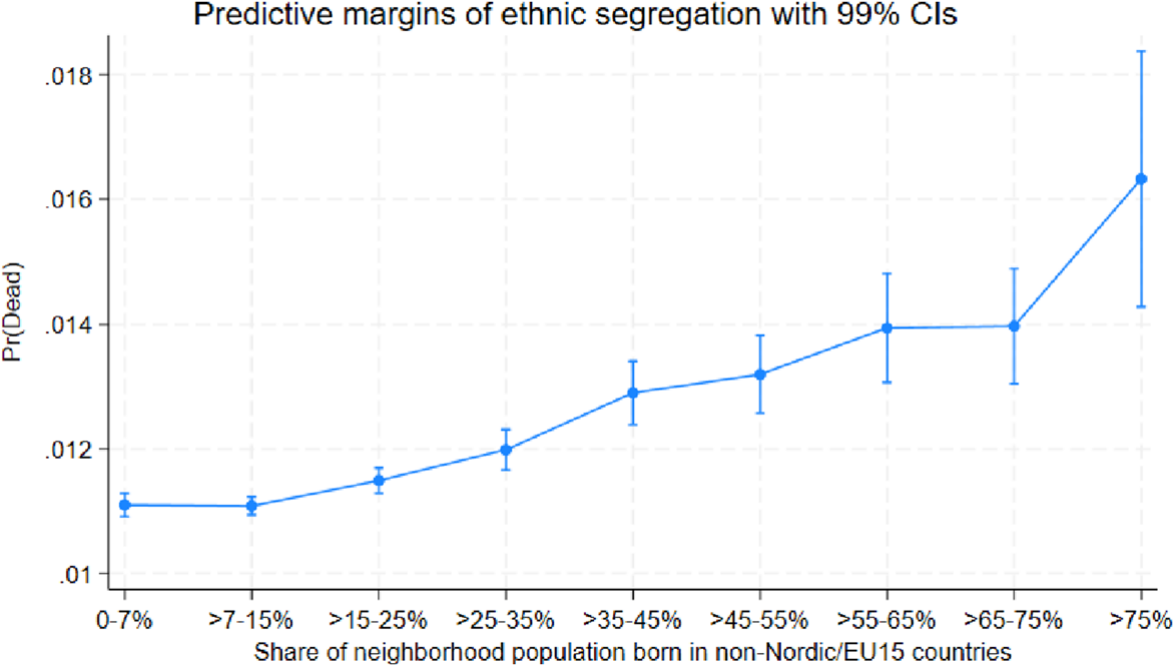
Predicted probability of death in various types of neighborhoods, categorized according to share of (non-Nordic/EU15) immigrants.

**Figure 5:**
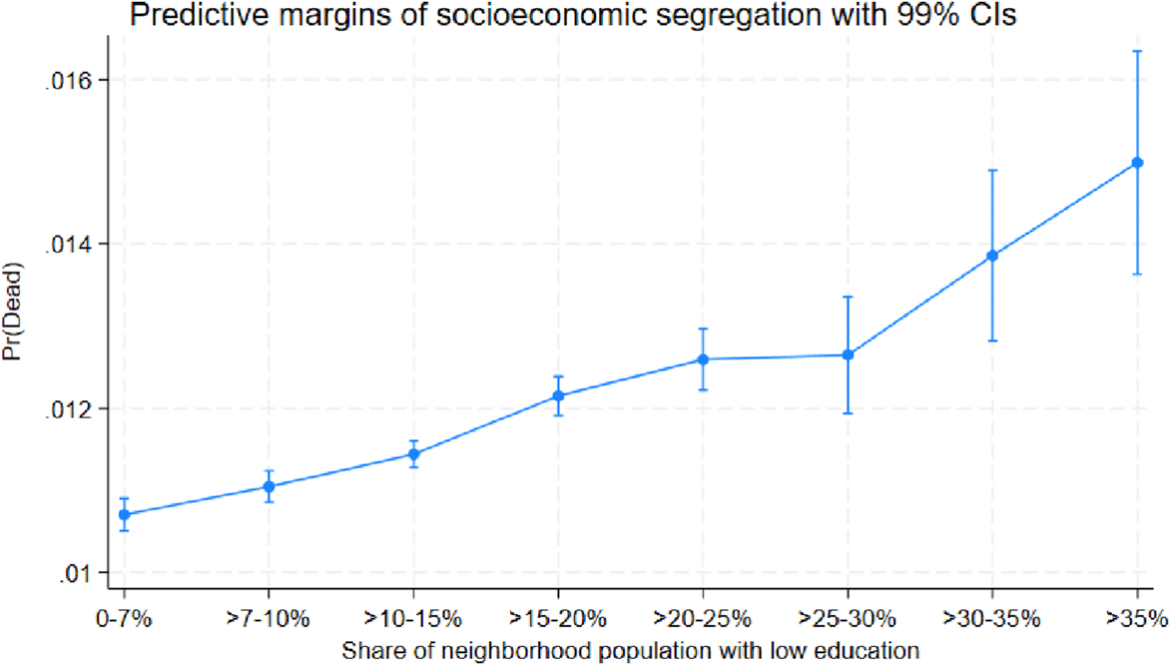
Predicted probability of death in various types of neighborhoods, categorized according to share of low educated population.

**Figure 6:**
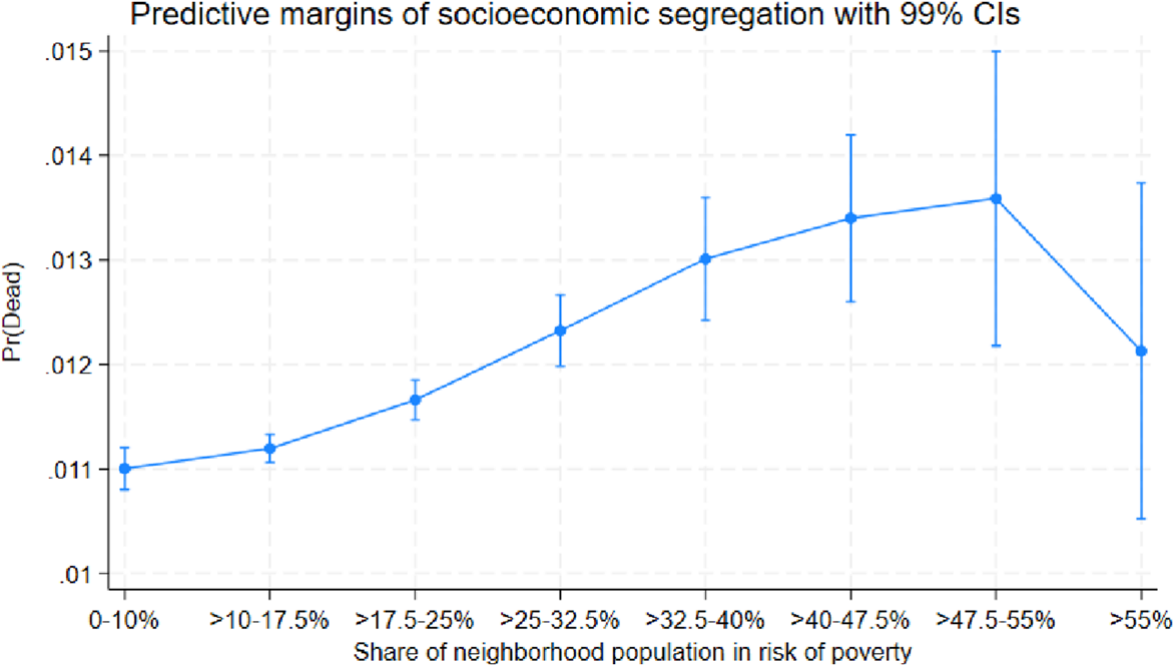
Predicted probability of death in various types of neighborhoods, categorized according to share in risk of poverty.

The marginal effects for disadvantaged neighborhoods reported in Table 1 may seem relatively small. For example, individuals residing in the neighborhoods with the highest shares of low educated (> 35%) are 0.43 percentage points more likely to die than individuals residing in the neighborhoods with the lowest shares of low educated (0–7%). To illustrate that this difference is not only statistically significant but also non-negligible from a socioeconomic perspective, Figs. 4, 5 and 6 show the predicted probability of dying for each socioeconomic category and neighborhood group. These figures illustrate how the probability to die increases as a function of the share of vulnerable individuals, for example, Fig. 4 shows that the probability to die is almost 50 percent higher in the most ethnically segregated neighborhoods (> 35% non-Nordic/EU15 immigrants), as compared to the least ethnically segregated neighborhoods (0–7% non-Nordic/EU15 immigrants). The predictive probabilities shown in Figs. 4, 5 and 6 are generated from the average marginal effects estimated for Table 1.

Table 2 shows the marginal effects of each of these local environment characteristics on the likelihood of COVID-19 death for an individual in each type of neighborhood. Note that the size of the estimates in Table 2 should not be compared to the estimates in Table 1 since the baseline probability of dying with COVID-19, given death, is greater than the overall probability of dying.

**Table 2:** Marginal effects for the probability of dying with COVID-19 at individual level, given that the individual has died (outcome equations).

	(1b)		(2b)		(3b)	
	i) Ethnic		ii) Low education		iii) Poverty	
Neighborhood characteristics						
	> 7–15%	.0047	> 7–10%	− .0032	> 10–17.5%	− .0008
	> 15–25%	.0053	> 10–15%	− .0064*	> 17.5–25%	− .0061*
	> 25–35%	.0010	> 15–20%	− .0093*	> 25–32.5%	− .0049
	> 35–45%	.0033	> 20–25%	− .0073*	> 32.5–40%	− .0010
	> 45–55%	− .0014	> 25–30%	.0023	> 40–47.5%	.0057
	> 55–65%	− .0001	> 30–35%	− .0039	> 47.5–55%	− .0103
	> 65–75%	.0069	> 35%	.0061	> 55%	.0296*
	> 75%	.0093				
No-go-zone	− .0067		− .0074*		− .0081*	
Regional characteristics						
Virus exposure	.0127*		.0126*		.0128*	
Population density	− .0039*		− .0044*		− .0043*	
Stockholm	.0349*		.0351*		.0366*	
Individual characteristics						
Ethnic background						
Nordic	.0016		.0020		.0018	
EU15	.0117*		.0118*		.0117*	
West Balkan	.0148*		.0150*		.0143*	
East Europe	.0173*		.0174*		.0170*	
Middle East	.0608*		.0610*		.0603*	
East Africa	.0868*		.0857*		.0863*	
North–South-West Africa	.0519*		.0511*		.0519*	
South-Central Asia	.0598*		.0599*		.0601*	
Southeast-East Asia	.0501*		.0499*		.0505*	
South-Central America	.0517*		.0514*		.0516*	
North America-Oceania	.0116		.0121		.0117	
Education						
High school	.0132*		.0129*		.0132*	
Shorter higher education	.0266*		.0259*		.0266*	
Longer higher education	.0299*		.0289*		.0300*	
Income	.0074*		0.0072*		0.0073*	
Age						
30–39	− .0014		− .0014		− .0014	
40–49	− .0083*		− .0083*		− .0085*	
50–59	− .0206*		− .0205*		− .0207*	
60–69	− .0476*		− .0472*		− .0477*	
70–79	− .0835*		− .0829*		− .0836*	
80–89	− .1566*		− .1559*		− .1568*	
90 +	− .2763*		− .2756*		− .2765*	
Female	.0291*		.0291*		.0292*	
Civil status						
Single with child	.0152		.0164		.0149	
Married	.0261*		.0261*		.0261*	
Married with child	.0614*		.0609*		.0612*	
Frontline worker	.0209*		.0213*		.0214*	
Elderly home	− .1239*		− .1241*		− .1243*	
House type						
Tenant-owned (apartment)	.0171*		.0167*		.0169*	
Owner-occupied (house)	.0209*		.0195*		.0188*	
Inter-generational household	.0049		.0049		.0049	
Crowded household	− .0003		− .0005		− .0007	
Multiple causes of death						
Two causes	.0676*		.0671*		.0679*	
Three causes	.0961*		.0954*		.0965*	
Four causes	.1165*		.1157*		.1169*	
Five or more causes	.1271*		.1261*		.1274*	
Diabetes	− .0099*		− .0099*		− .0098*	
Observations	97,197		97,197		97,197	
Wald Chi2	44,901*		45,447*		45,037*	

*Notes:* Dependent variable = 1 if dead in COVID-19, zero if other cause/s of death. * denotes significance at 1% level.

Surprisingly perhaps, we see that the general effects of distinct neighborhood characteristics – whether ethnicity, education attainment or incomes levels—on individual risk of dying with COVID-19 are statistically insignificant in 17 out of 22 cases. In other words, once we control for individual and locational characteristics associated with higher likelihood of death from all causes, we find little or no support that any neighborhood characteristics moderate the proportion of dead dying with COVID-19 specifically. The richness and completeness of the data for Sweden allows us to discuss these effects in great detail.

We find a positive and significant result for COVID-19 mortality for neighborhoods with lower shares of foreign born (> 7–15% and > 15–25% in Table 1), and also some support for the educational level effect as individuals in more educated neighborhoods (with up to 10–25% share of less educated) were *less* likely to die. Most importantly, we find no significant results for an increased risk of COVID-19 deaths in the most extreme communities in terms of education or ethnicity (> 7–10% nor > 35%), so all effects are found at more intermediate levels of mixing. These more extreme communities comprise of the most marginalized neighborhoods or the neighborhoods that are most affluent. In terms of income, we find a higher marginal effect on the COVID-19 share of mortality for the highest poverty neighborhoods (> 55%). Further analysis shows that several of these neighborhoods are student areas in college towns (that is, neighborhoods where many university students reside) with a low share of non-Nordic/EU immigrants and a low share of the less educated. These neighborhoods are not considered marginalized, which explains their lower and insignificant marginal effect on the probability for overall death (selection equation). On the other hand, there have been reports of COVID-19 outbreaks among specific student groups (for example, in the university town of Uppsala), which implies that students may have contributed to a greater spread of infection into the local general population. Indeed, the results of the outcome equation show that individuals residing in student areas have a 3% higher probability of dying with COVID-19 than from other causes. These deaths are not infected students, but rather the more vulnerable and/or older individuals living in the same areas, as we discuss below in terms of age profiles.

At the *individual level* (see “Individual characteristics”), it is interesting to note the negative significant results for older age groups relative to dying with COVID-19 (Table 2). While this may sound counterintuitive, it is simply the result of the selection bias that we correct for in Step 1 of the analysis (Table 1). Older persons are more likely to die to start with (general causes) but, given death at an advanced age, are actually *less* likely to have COVID-19 as their cause of death. For individuals who are foreign-born, we find an opposite pattern. Individuals with a non-Swedish ethnic background are in most cases less likely to die from general causes (Table 1), but among those who died, the cause of death is *more likely* to be COVID-19 (Table 2). The ethnicity most at risk in our analysis is East African, showing the highest marginal effect: approximately 7 times higher than for individuals from EU15. These results are in line with previous studies on COVID-19 deaths across ethnic groups.

As expected, we also find a higher probability of dying with COVID-19 the more deleterious health conditions an individual has. This confirms clinical observations that people with multiple serious conditions are at a greater risk of becoming fatally ill from a COVID-19 infection. There is a significant marginal effect of up to 13 percentage points for individuals with five or more causes of death. On the other hand, after controlling for all other factors, individuals diagnosed with diabetes are less likely to die with COVID-19, but the effect is small since the decrease in the probability of dying with COVID-19 is less than one percentage point. Since diabetes has been brought up as a risk factor in COVID-19 deaths, our results may appear counterintuitive. They may however be explained by the fact that diabetes patients often suffer from several potentially life-threatening conditions^64^, and the risk of diabetes may thus be captured by the effect of multiple causes of death. Indeed, when excluding multiple causes of death diabetes turns positive although insignificant.

Regarding regional characteristics, individuals residing in Stockholm (the Swedish capital) and those exposed to a greater number of COVID-19 infected in their municipality have a higher probability of having COVID-19 as a cause of death. On the other hand, the results on population density are significantly negative, which goes against the expectations and is likely due to collinearity with the variable on virus exposure. Indeed, when excluding virus exposure population density turns significantly positive, despite a less than 0.5 bivariate correlation between the variables. The remaining variables are robust to the exclusion of virus exposure.

Taken together, we find little support for neighborhood effects mediated by socioeconomic segregation on the likelihood of dying with COVID-19, but strong support that individual-level characteristics, especially related to specific ethnic backgrounds, may have played a role.

### Statistical analysis by age groups and urban areas

To further investigate other important effects, we focus our estimation procedures on more specific subgroups since (1) the majority of deaths are in the age group 70 and older, and (2) the role of neighborhoods may matter differently in urban versus rural settings. Our objective with this more disaggregated analysis is to investigate possible confounding results due to averaging. For example, it may be the case that the selection bias estimation (step 1) generates a different result if we target specific groups instead of comparing the probability of dying for the whole population. Starting with an analysis where we only include individuals aged 70 and over, Tables 3 and 4 present the average marginal effects for neighborhood characteristics (full tables are available from the authors upon request). As noted, 90% of deaths with COVID-19 in Sweden belong to this age group. The number of observations decreases relatively more in the selection equations (compare Table 3 with Table 1) than in the outcome equations (compare Table 4 with Table 2), reflecting the higher probability of dying in the older age group (70 +) than in the overall population (15 +).

**Table 3:** Marginal effects for the probability of dying at individual level, aged 70 and above (selection equations).

	(4a)		(5a)		(6a)	
	i) Ethnic		ii) Low education		iii) Poverty	
Neighborhood characteristics						
	> 7–15%	− .0007	> 7–10%	.0014*	> 10–17.5%	.0011
	> 15–25%	.0008	> 10–15%	.0026*	> 17.5–25%	.0020*
	> 25–35%	.0030*	> 15–20%	.0046*	> 25–32.5%	.0045*
	> 35–45%	.0075*	> 20–25%	.0058*	> 32.5–40%	.0072*
	> 45–55%	.0065*	> 25–30%	.0066*	> 40–47.5%	.0100*
	> 55–65%	.0115*	> 30–35%	.0146*	> 47.5–55%	.0149*
	> 65–75%	.0118*	> 35%	.0171*	> 55%	.0081
	> 75%	.0297*				
Control variables	YES		YES		YES	
Observations	1,524,203		1,524,203		1,524,203	
Wald Chi2	35,417*		34,644*		34,643*	

*Notes:* Dependent variable = 1 if dead, zero otherwise. * denotes significance at 1% level.

**Table 4:** Marginal effects for the probability of dying with COVID-19 at individual level, given that the individual has died, aged 70 and above (outcome equations).

	(4b)		(5b)		(6b)	
	i) Ethnic		ii) Low education		iii) Poverty	
Neighborhood characteristics						
	> 7–15%	.0055*	> 7–10%	− .0040	> 10–17.5%	− .0010
	> 15–25%	.0059	> 10–15%	− .0060*	> 17.5–25%	− .0048
	> 25–35%	.0000	> 15–20%	− .0088*	> 25–32.5%	− .0028
	> 35–45%	− .0009	> 20–25%	− .0057	> 32.5–40%	− .0026
	> 45–55%	− .0003	> 25–30%	.0062	> 40–47.5%	.0049
	> 55–65%	− .0009	> 30–35%	− .0153	> 47.5–55%	− .0131
	> 65–75%	.0073	> 35%	.0009	> 55%	.0223
	> 75%	− .0052				
Control variables	YES		YES		YES	
Observations	80,898		80,898		80,898	
Wald Chi2	35,417*		34,644*		34,643*	

*Notes:* Dependent variable = 1 if dead with COVID-19, zero if dead from other cause/s. * denotes significance at 1% level.

We find that neighborhood characteristics did not add any significant risk to the likelihood of dying with COVID-19 for the oldest age groups and, specifically, that neither ethnically nor educationally segregated neighborhoods add to this risk. However, we do find a weakly significant (10 percent level) increased risk for elderly individuals who live in neighborhoods where more than 55% of the population is categorized as poor. Recall that these are primarily student residential areas in college towns. The elderly may have been adversely affected by what was reported in the media as an increased spread of COVID-19 resulting from student gatherings and lack of compliance with social distancing recommendations in several of these towns during the pandemic. While the students themselves may not have been at any severe risk of death, we find that the elderly residing in these neighborhoods are more likely to die specifically with COVID-19.

We also performed the estimation analysis separately for urban and rural regions since neighborhood effects may play out differently depending on this type of context. For example, recent work indicates that bigger, more urban regions also tend to be more segregated^65,66^. We also ran a separate analysis for Stockholm, Sweden’s capital city, as well as for the metropolitan regions of Stockholm, Gothenburg, and Malmö combined (metropolitan regions being the equivalent of spatially extended labor markets). Table 5 and 6 present the average marginal effects for the neighborhood segregation variables for metropolitan municipalities, urban municipalities, and rural municipalities, respectively. The separate estimation for individuals residing in the Stockholm region includes 19 municipalities classified as metropolitan plus six urban municipalities and one rural municipality.

**Table 5:** Marginal effects of the neighborhood characteristics for the probability of dying at individual level, per region type (selection equations).

	7a	8a	9a	10a
	Stockholm	Metro	Urban	Rural
i) Ethnic				
> 7–15%	.0003	.0005	-.0002	.0002
> 15–25%	.0007	.0009*	.0002	.0010*
> 25–35%	.0013*	.0014*	.0008*	.0000
> 35–45%	.0018*	.0023*	.0014*	.0020
> 45–55%	.0017*	.0021*	.0025*	.0029
> 55–65%	.0030*	.0029*	.0023*	.0065
> 65–75%	.0024*	.0027*	.0039*	
> 75%	.0040*	.0042*	.0064*	
ii) Low education				
> 7–10%	.0006*	.0005*	.0001	.0008
> 10–15%	.0009*	.0011*	.0004*	.0010
> 15–20%	.0016*	.0018*	.0013*	.0014
> 20–25%	.0021*	.0020*	.0016*	.0025*
> 25–30%	.0031*	.0027*	.0013*	.0019
> 30–35%	.0028*	.0026*	.0038*	.0003
> 35%	.0053*	.0030*	.0048*	.0070*
iii) Poverty				
> 10–17.5%	.0005*	.0004*	− .0000	.0006
> 17.5–25%	.0014*	.0011*	.0004	.0008
> 25–32.5%	.0021*	.0014*	.0010*	.0019*
> 32.5–40%	.0015*	.0017*	.0020*	.0035*
> 40–47.5%	.0025*	.0022*	.0022*	.0023
> 47.5–55%		.0018*	.0033*	
> 55%	− .0041	− .0007	.0035*	
Control variables	YES	YES	YES	YES
Observations	1,930,775	2,817,997	4,257,429	1,403,590

*Notes:* As for Table 4 above, the estimations including *i) Ethnic*, *ii) Low education*, and *iii) Poverty* (to describe neighborhoods) are run separately. All control variables included. * denotes significance at 1% level.

**Table 6:** Marginal effects of the neighborhood characteristics for the probability of dying with COVID− 19 at individual level, given that the individual has died, per region type (outcome equations).

	7b	8b	9b	10b
	Stockholm	Metro	Urban	Rural
i) Ethnic				
> 7–15%	− .0080	− .0065	.0059*	− .0022
> 15–25%	− .0102	− .0074	.0054*	− .0015
> 25–35%	− .0130*	− .0118*	.0005	.0024
> 35–45%	− .0122	− .0163*	.0030	.0019
> 45–55%	− .0143*	− .0136*	− .0059	− .0125
> 55–65%	− .0202*	− .0167*	.0016	− .0915
> 65–75%	− .0087	− .0095	− .0080	
> 75%	− .0060	− .0120	.0057	
ii) Low education				
> 7–10%	− .0051	− .0046	− .0008	.0030
> 10–15%	− .0075*	− .0109*	− .0006	− .0058
> 15–20%	− .0085*	− .0113*	− .0062*	− .0074
> 20–25%	− .0147*	− .0133*	− .0024	− .0112
> 25–30%	− .0188	− .0093	− .0003	.0011
> 30–35%	− .0012	− .0080	− .0116	− .0100
> 35%	− .0071	− .0005	− .0057	− .0289
iii) Poverty				
> 10–17.5%	− .0053*	− .0036	.0005	− .0037
> 17.5–25%	− .0121*	− .0090*	− .0024	− .0072
> 25–32.5%	− .0147*	− .0104*	− .0037	− .0065
> 32.5–40%	− .0082	− .0091	− .0031	− .0152
> 40–47.5%	.0028	− .0022	− .0039	− .0218
> 47.5–55%		− .0131	− .0166	
> 55%	− .2839*	.0124	.0064	
Control variables	YES	YES	YES	YES
Observations	18,196	26,350	50,458	20,389

*Notes: *As for Table 3 above, the estimations including *i) Ethnic*, *ii) Low education*, and *iii) Poverty* (to describe neighborhoods) are run separately. All control variables included. * denotes significance at 1% level.

Table 5 summarizes these results showing that, regardless of regional type, we observe that more segregated neighborhoods in terms of ethnicity, education levels, and poverty increase the marginal effects of the probability of dying from all causes. The exception is rural regions, with fewer significant but still positive coefficients. As per Table 6, living in marginalized neighborhoods, in this sense, does not add significantly to the risk of dying specifically with COVID-19. There are some significant estimates however, in particular for Stockholm and metropolitan municipalities and individuals there residing in neighborhoods with an intermediate share of foreign-born, low educated, and poor. However, the signs are negative, indicating that individuals residing in these neighborhoods are less likely to die with COVID-19 than individuals residing in the least ethnically segregated and socioeconomically strongest neighborhoods.

## Discussion and conclusions

The COVID-19 pandemic brought to the forefront of both public discussions and scientific research inequalities in the burden of disease and mortality, especially affecting marginalized local communities. Much research reported so far, found excessive deaths in ethnically segregated and socioeconomically disadvantaged local communities^67,68^. There are also several previous empirical studies connecting COVID-19 deaths to individual and neighborhood characteristics. However, these studies have not adjusted for the underlying selection bias resulting from spatial variations in general mortality.

The most ambitious studies have looked at the likelihood of dying given infection^69^, but not if this is a group that is likely to die to start with. A typical approach has been to examine “excess mortality” but at the national level, not at the individual level^70^. Here, we used a full-population, geocoded micro-dataset for Sweden to analyze in detail if and how the characteristics of residential neighborhoods affected the likelihood of dying with COVID-19 after controlling for a rich set of individual characteristics and comparing with background death rates for the same groups in Sweden during 2020. The scope of our datasets and the design of our statistical approach allow us therefore to account explicitly for selection bias reflecting the probability of overall death by all causes, which proved critically important in our findings. At the same time, our two-step modelling approach, which is unique in studies on COVID-19 deaths, also comes with the limitation that our estimates are not directly comparable to previous studies on this topic. Nevertheless, we argue that to understand and to not overestimate the role played by residential contexts during the pandemic, it is necessary to account for individual and geographic heterogeneity in the general risk of dying.

Our results show that neighborhood characteristics played at best an extremely marginal role in explaining the probability of dying with COVID-19 in Sweden. Neither ethnicity nor lower levels of education at the neighborhood scale add to the likelihood of dying with COVID-19. On the other hand, we do observe an increased risk of COVID-19 deaths in neighborhoods with a high proportion of residents categorized as “poor." On closer inspection, however, we found that these neighborhoods are mainly student residential areas in college towns, not classically considered as marginalized communities. In these local contexts, we show that high COVID-19 death rates are the result of specific population mixing patterns, where a less compliant young population in terms of social distancing interacts with an older set of individuals, who in turn bear the brunt of mortality. In other words, there is little evidence from our full population study that there are neighborhood effects connected to segregation or disadvantage mediating the probability of dying with COVID-19 in Sweden. Observed variations can be explained by differences of individual characteristics, where, e.g., specific ethnicity plays a significant role.

There may be several reasons why ethnicity influenced the probability of dying during the pandemic in the context of how Swedish authorities responded to the pandemic. Instead of relying on a mandatory lockdown, the Swedish COVID-19 strategy was based on the assumption that individuals would follow the recommendations of public health authorities^71^. International surveys, such as Inglehart's World Value Survey, show that residents of Sweden generally have high levels of trust in their authorities and in researchers. This trust in government experts in turn was taken to imply trusting public health advice^72–74^. Studies of migrant groups in Sweden, however, indicate that specific groups can have significantly lower levels of trust in public authorities^75^. As a result, information about what to do in response to the pandemic might have been obtained from sources in the migrants’ countries of origin or through informal channels within their own networks. Swedish medical doctors raised concerns that this strategy would have been better suited for a more homogeneous society^76^. Contrary to prevalent views about Sweden, the country is significantly heterogeneous, with 20% of the resident population being foreign-born. Language may also have been a barrier as the initial contact with Swedish healthcare often takes place by telephone and some individuals may have been limited in their ability to describe their medical situation. Further, there could be cultural differences that affect the extent to which one seeks care for different types of symptoms^77^. Studies have also shown how immigrants in Sweden are three to four times more likely than native Swedes to say they suffer from poor or very poor health, often due to factors related to place of origin, the refugee process, as well as the challenge of being new to a country and in a socially vulnerable situation^78^. Foreign born individuals are also less likely to use health screening programs^79,80^. Female foreign-born individuals from some countries are also more likely to suffer from obesity than native Swedish women^81^.

We did find, however, that neighborhood characteristics play a clear and important role in the general likelihood of death from all causes both pre- and during the pandemic. Individuals living in marginalized areas display a significantly higher risk of dying, whatever their individual characteristics. Reconciling these two results means that this elevated likelihood of death is shared among all causes and is not specific to COVID-19. A major conclusion from these results is that the role of neighborhood and individual vulnerability—captured here by the risk of death—is in part “ecological”, in that it cannot be fully explained by individual characteristics.

It is important to note that Sweden has an extensive public (i.e., free) healthcare system. It is organized at the provincial level (“regions” in Swedish) and divided into two levels: primary and secondary care. Primary care consists of slightly over 1000 health centers distributed throughout the country. This is normally the first contact made with healthcare. If necessary, the patient is referred to secondary or more specialized tertiary care, which consists mainly of hospitals that cover larger geographical areas. It could be the case that health centers in marginalized neighborhoods were unable to provide care of the same quality as the ones located in better off neighborhoods. It has been documented that health disparities are greater within regions than between them, and that this has a negative effect on the disadvantaged groups in society^82^. Furthermore, the work situation at health centers in marginalized neighborhoods is reported to be more challenging as the need is often greater, but resources are limited^83^. Even though Swedish healthcare is free and of a high quality by international standards, there are still reports of discrimination against certain groups when it comes to treatment and a lower probability of gaining access to certain types of care^84^.

Although the datasets available for Sweden are special in terms of their details and completeness, our statistical procedures and findings should be replicable in other nations with population registries, such as other Scandinavian nations, the Netherlands or Austria, as well as in other contexts where detailed death records are available. The hypothesis advanced by our study for these contexts is that apparent elevated burdens of mortality with COVID-19 fall along the same statistical lines as burdens of general mortality at the individual and community levels, once these specific contexts can be accounted for. We encourage further studies to test this hypothesis beyond the case of Sweden to allow for cross-country comparisons.

A limitation of our study following the official definition of COVID-19 deaths in Sweden is however that an individual passing away within 30 days after a confirmed positive COVID-19 test is classified as a COVID-19 death even though the death may have been primarily due to other causes. Even though we do control for multiple causes of death, this definition may bias our estimates of neighborhood effects on COVID-19 deaths somewhat, most likely downwards. For future studies a more detailed coding of deaths from COVID-19 (and other infectious virus diseases) by the Swedish National Board of Health and Welfare would be beneficial. Analyses of determinants of mortality once vaccination became available in 2021 are also an important theme for future studies.

The results presented here might have generalizable and practical policy implications, specifically in contributing methods to better assess the social determinants of health and emphasizing the importance of holistic approaches to human development. They show that statistical analyses of health inequalities in marginalized neighborhoods conducted in connection with the COVID-19 pandemic must be examined in broad terms, beyond the analysis of a singular emergency. In our study, COVID-19 appears as a new stress creating additional mortality along statistically engrained population risk patterns, expressed as systemic community level dynamics affecting all mortality. These findings suggest therefore that while emergencies such as the COVID-19 pandemic necessarily drive attention and policy over the short term, there might be systemic factors that need be better understood and mitigated over the longer term associated with higher risk of death in marginalized neighborhoods. These factors cannot be explained by an individual’s age, income, or education. While the COVID-19 pandemic has highlighted the vulnerability of marginalized neighborhoods at a time of crisis, we are learning that the best defense against the next pandemic may well be the systemic improvement of health conditions in disadvantaged communities at all times.

### Supplementary Information

Supplementary Information.

## Data Availability

This study does not involve direct human participants. Instead, it is based on anonymized register data. These data are available from Statistics Sweden (SCB, available at: https://www.scb.se/en/services/ordering-data-and-statistics/ordering-microdata/) and the National Board of Health and Welfare (Socialstyrelsen, available at: https://bestalladata.socialstyrelsen.se/data-for-forskning/sekretessprovning/) following an ethical review from the Swedish Ethical Review Authority (Swe: Etikprövningsmyndigheten, available at: https://etikprovningsmyndigheten.se/) and a review of secrecy. This study was approved by the Swedish Ethical Review Authority with diary numbers 2018/174–31 and 2020–05,497 and carried out in accordance with relevant guidelines and regulations. The data in the figures is available via https://github.com/mansueto-institute/Neighborhood-Effects-on-COVID-Fatalities-in-Sweden.
