# Publisher Correction: Evidence of COVID-19 fatalities in Swedish neighborhoods from a full population study

**DOI:** 10.1038/s41598-024-57170-3

**Published:** 2024-03-21

**Authors:** Sofia Wixe, José Lobo, Charlotta Mellander, Luís M. A. Bettencourt

**Affiliations:** 1 Centre for Entrepreneurship and Spatial Economics, Jönköping International Business School, Jönköping University, Jönköping, Sweden; 2 School of Sustainability, College of Global Futures, Arizona State University, Tempe, AZ USA; 3 Mansueto Institute for Urban Innovation, University of Chicago, Chicago, IL USA; 4 Department of Ecology & Evolution, Department of Sociology, University of Chicago, Chicago, IL USA

Correction to: *Scientific Reports* 10.1038/s41598-024-52988-3, published online 06 February 2024

In the original version of this Article Charlotta Mellander was incorrectly affiliated with ‘Mansueto Institute for Urban Innovation, University of Chicago, Chicago, IL, USA’. The correct affiliations are listed below.

Centre for Entrepreneurship and Spatial Economics, Jönköping International Business School, Jönköping University, Jönköping, Sweden.

The original Article has been corrected.

