# Supplementary material for: Evidence of COVID-19 fatalities in Swedish neighborhoods from a full population study
Source: Sci Rep. 2024 Feb 6;14:2998. doi: 10.1038/s41598-024-52988-3 (PMC10844299; doi:10.1038/s41598-024-52988-3)
Supplement: Supplementary file 1 — Supplementary Information. [file 41598_2024_52988_MOESM1_ESM.docx]

Supplementary Information for

**Neighborhoods and COVID-19 Fatalities: Evidence from a full population study in Sweden**

**Sofia Wixe^1^, José Lobo^2^, Charlotta Mellander^3^* Luís M.A. Bettencourt^4,5^**

*Corresponding author. Email: [charlotta.mellander@ju.se](mailto:charlotta.mellander@ju.se)

**This file includes:**

The Supplementary Material Figures S1-S7 present descriptive statistics for all neighborhood variables. Tables S1-S3 present descriptive statistics for the individual level descriptive statistics variables. Table S4 illustrates what countries are included in each region of origin.

**Other Supplementary Materials for this manuscript include the following:**

Data Figures S1-S7, Tables S1-S4


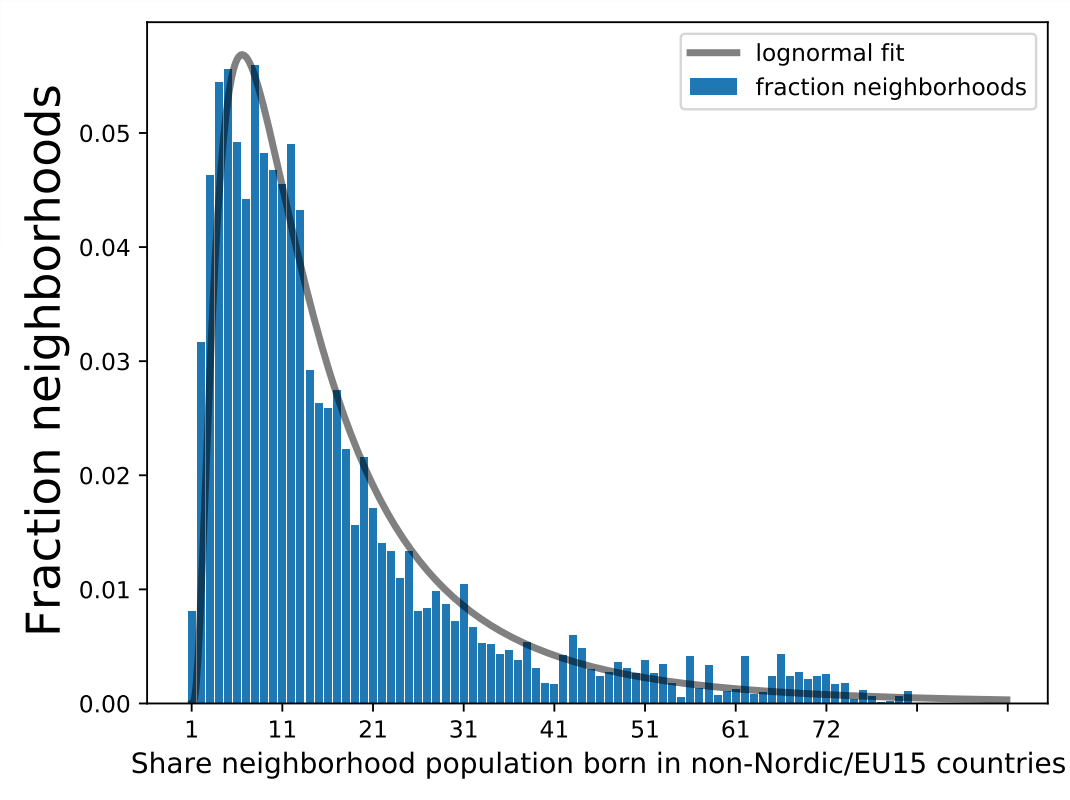


**Figure S1.** Distribution of individuals across different neighborhoods, according to share of population born in non-Nordic/EU15 countries (*Ethnic segregation*).


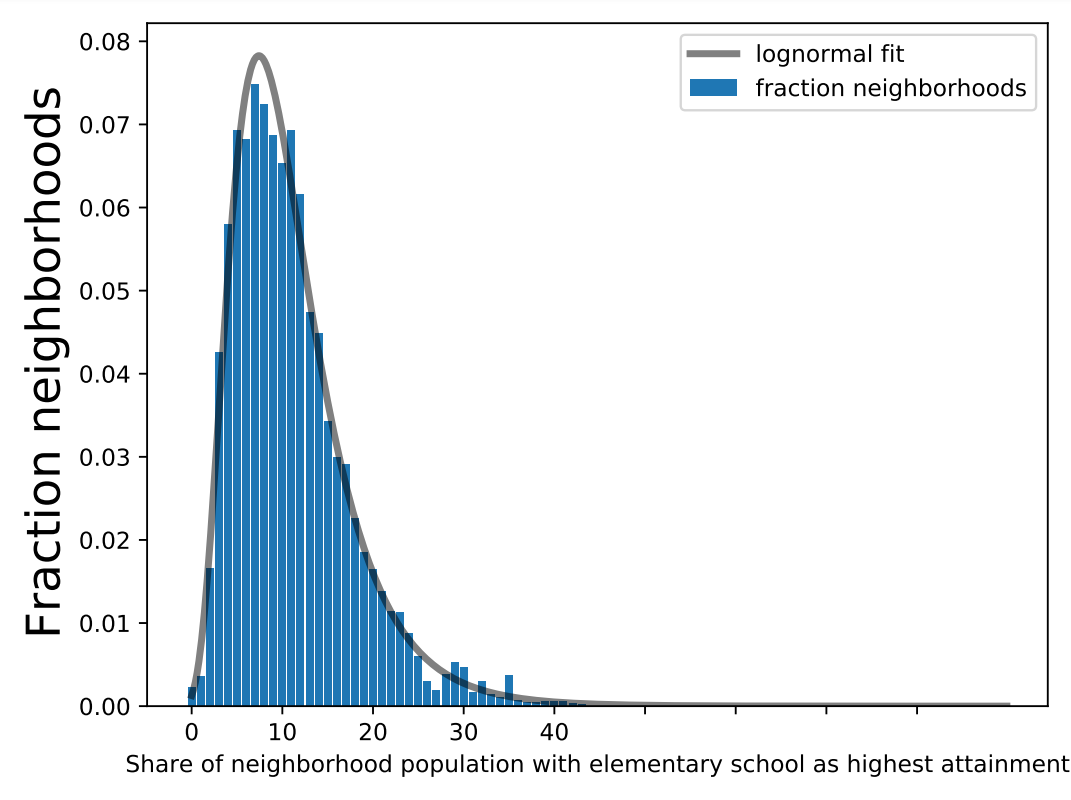


Figure S2. Distribution of individuals across different neighborhoods, according to share of population (aged 20-64) with elementary school as highest educational attainment (*Socioeconomic segregation – Low education*).


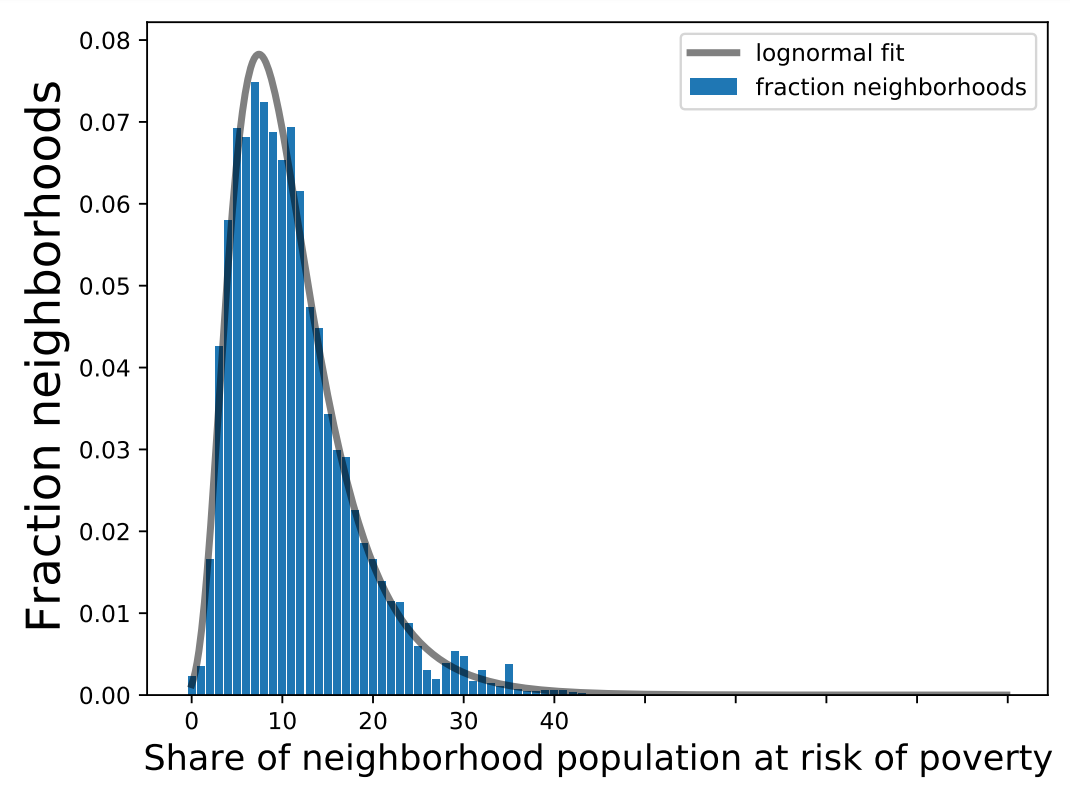


Figure S3. Distribution of individuals across different neighborhoods, according to share of population (aged 20+) in risk of poverty (*Socioeconomic segregation – Poverty*).


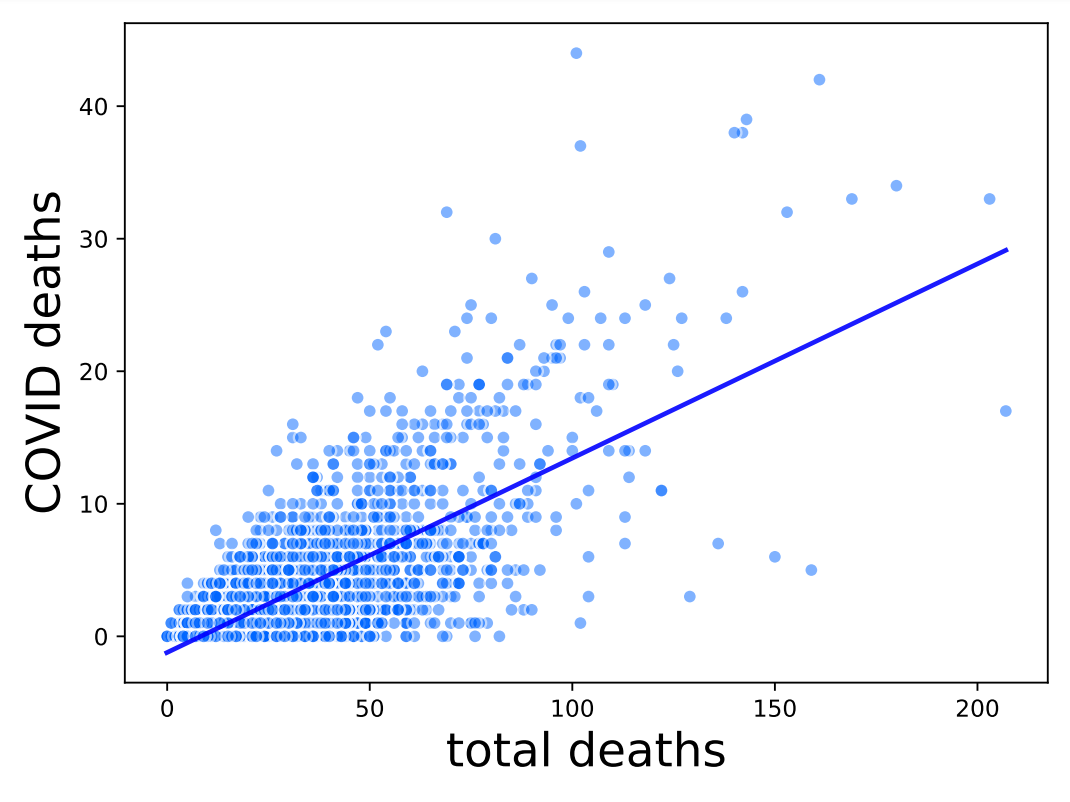
**Figure S4.** Total COVID-19 deaths vs all deaths per neighborhood.


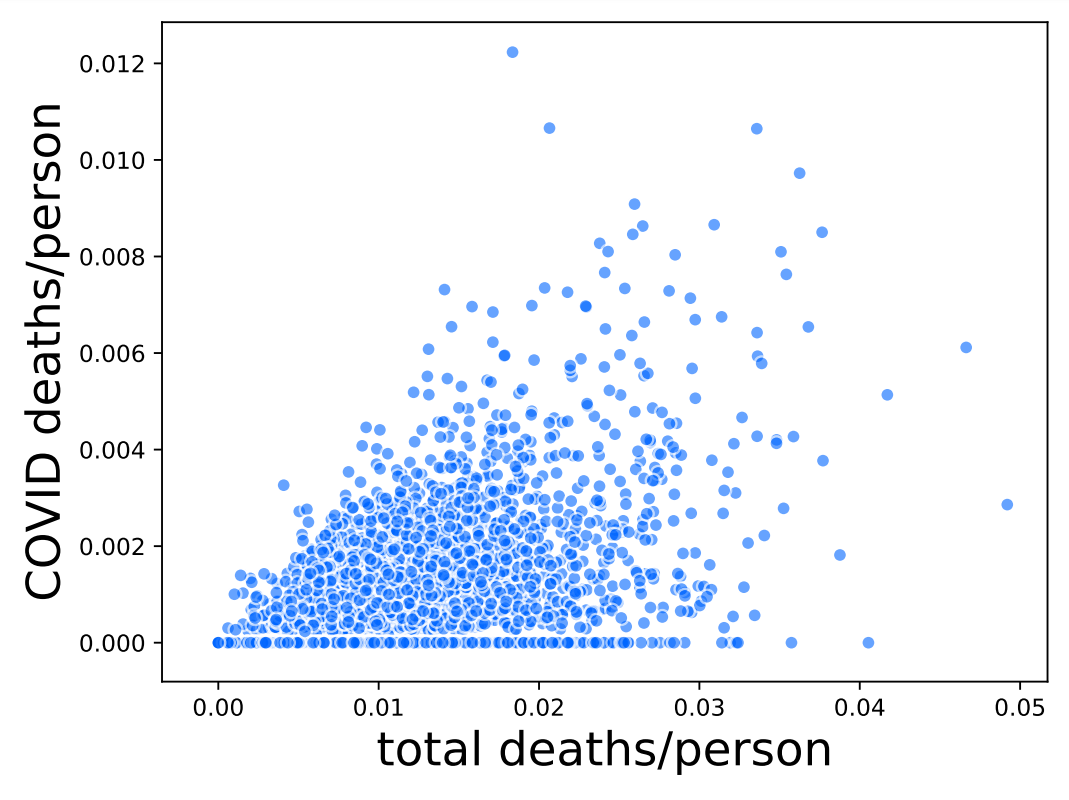


Figure S5. Total COVID-19 deaths vs all deaths per neighborhood, both weighted by neighborhood population.


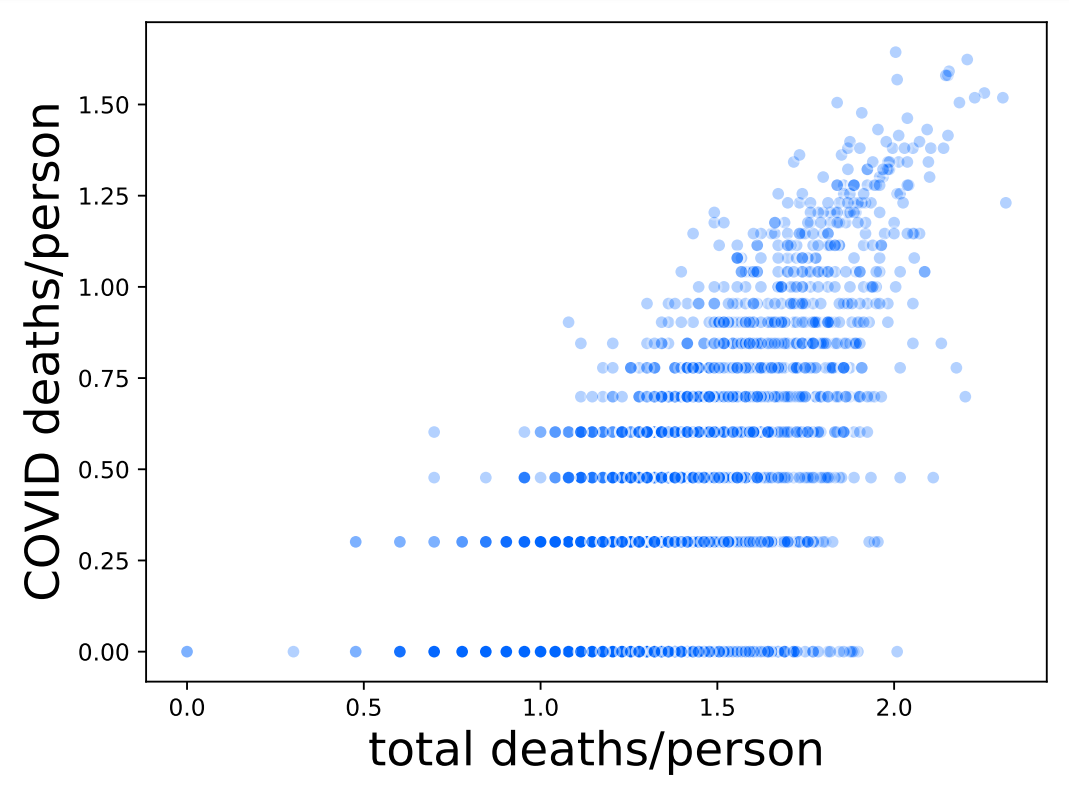


Figure S6. Total COVID-19 deaths vs all deaths per neighborhood, both log transformed. Neighborhoods with zero COVID-19 deaths are excluded.


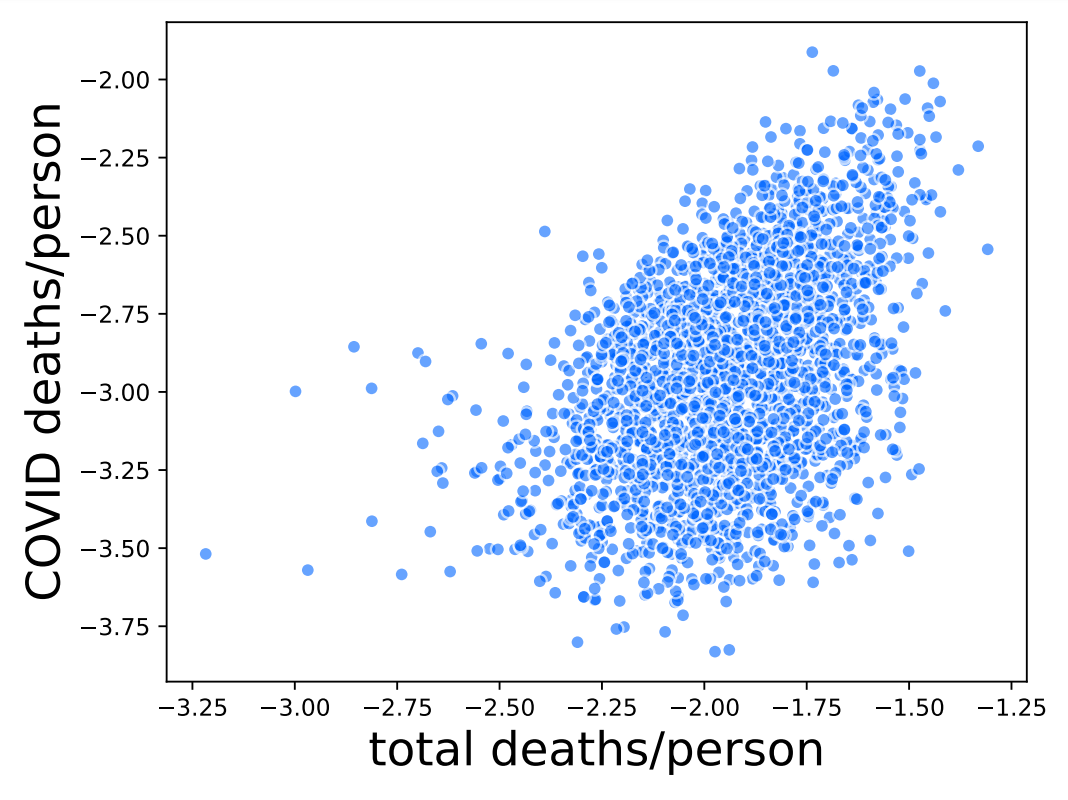


Figure S7. Total COVID-19 deaths vs all deaths per neighborhood, both weighted by neighborhood population, both log transformed. Neighborhoods with zero COVID-19 deaths are excluded.

Table S1. Variables Description.

| **Variable** | **Equation** | **Description** | | | | | | |
| --- | --- | --- | --- | --- | --- | --- | --- | --- |
| Dead | Selection (S) | Binary variable equal to one if individual *i* died in year 2020, zero otherwise. Dependent variable in the selection equation. | | | | | | |
| COVID-19 | Outcome (O) | Binary variable equal to one if individual *i* died with COVID-19 in year 2020, zero if the individual died by any other cause/s. Dependent variable in the outcome equation.  In Sweden, the definition of a COVID-19 related death includes both (1) a person reported dead by a medical doctor via SmiNet (the database system used to report by law notifiable infectious diseases) or by the health care sector via the regional infectious restraints, and (2) a person who has died within 30 days after a confirmed COVID-19 test has been taken. | | | | | | |
| *Neighborhood characteristics* |  |  | | | | | | |
| Ethnic | S + O | Categorical variable showing share of neighborhood population born in non-Nordic/EU15 countries. | | | | | | |
|  |  | - 0 – 7% (base)  - >7 – 15%  - >15 – 25%  - >25 – 35%  - >35 – 45% | | - >45 – 55%  - >55 – 65%  - >65 – 75%  - >75% | | | | |
| Low education | S + O | Categorical variable showing share of population aged 20-64 with elementary school as highest educational attainment. | | | | | | |
|  |  | - 0 – 7% (base)  - >7 – 10%  - >10 – 15%  - >15 – 20% | | | | - >20 – 25%  - >25 – 30%  - >30 – 35%  - >35% | | |
| Poverty | S + O | Categorical variable showing share of population aged 20 and above in risk of poverty, defined as having an equivalized disposable income below 60 percent of the corresponding national median (69). | | | | | | |
|  |  | - 0 – 10% (base)  - >10 – 17.5%  - >17.5 – 25%  - >25 – 32.5% | | | | | - >32.5 – 40%  - >40 – 47.5%  - >47.5 – 55%  - >55% | |
| No-go-zone | S + O | Binary variable equal to one if residing in a post code area classified as especially distressed by the Swedish police. | | | | | | |
| *Regional characteristics* |  |  | | | | | | |
| Population density | S + O | Natural logarithm of population per square kilometer per municipality. | | | | | | |
| Regional type | S | Categorical variable.  - Rural municipalities (base)  - Urban municipalities  - Metropolitan municipalities | | | | | | |
| Stockholm | O | Dummy equal to one if residing in Stockholm county (26 municipalities). | | | | | | |
| Virus exposure | O | Access to total number of confirmed COVID-19 cases in the municipality and county one week^[[1]](#footnote-1)^ before death (spatially weighted total number of cases in the county, accounting for travel time between municipalities)^[[2]](#footnote-2)^. Given that Sweden had no strict lockdown and individuals could move freely, one might have been exposed to the virus also outside of the own neighborhood. | | | | | | |
| *Individual characteristics* |  |  | | | | | | |
| Ethnic background | S + O | Categorical variable (see Table S5 for included countries in each category). | | | | | | |
|  |  | - Sweden (base)  - Nordic  - EU15  - West Balkan  - East Europe  - Middle East | - East Africa  - North-South-West Africa  - South-Central Asia  - Southeast-East Asia  - South-Central America  - North America-Oceania^[[3]](#footnote-3)^ | | | | |  |
| Education | S + O | Categorical variable showing highest formal schooling attainment.  - Elementary school (base)  - High school  - Shorter higher education (< 3 years)  - Longer higher education (> 3 years) | | | | | | |
| Income | S + O | The natural logarithm of disposable income, inferred from the whole family’s disposable income (disposable income per consumption weight). | | | | | | |
| Age | S + O | Categorical variable of age groups. | | | | | | |
|  |  | - 15 – 29 (base)  - 30 – 39  - 40 – 49  - 50 – 59 | | | - 60 – 69  - 70 – 79  - 80 – 89  - 90 + | | | |
| Female | S + O | Binary variable equal to one if female. | | | | | | |
| Civil status | S + O | Categorical variable.  - Single with no children (base)  - Single with at least one child below 18  - Married/co-habitant with no children  - Married/co-habitant with at least one child below 18 | | | | | | |
| Frontline worker | S + O | Binary variable equal to one if having an occupation classified as frontline, including health and welfare workers, police, cashiers, taxi- and public transport drivers. | | | | | | |
| Elderly home | S + O | Binary variable equal to one if residing in an elderly home. | | | | | | |
| House type | S + O | Categorical variable.  - Rental apartment (base)  - Tenant-owned apartment  - Owner-occupied house | | | | | | |
| Inter-generational household | O | Binary variable equal to one if residing in a household with at least one member aged below 18 and at least one member aged 70 or above. | | | | | | |
| Crowded household | O | Binary variable equal to one is residing in a household classified as crowded, excluding single households without children in one-room apartments^[[4]](#footnote-4)^. | | | | | | |
| Multiple causes | O | Categorical variable showing the number of unique causes of death.  - One cause of death (base)  - Two causes of death  - Three causes of death  - Four causes of death  - Five or more causes of death | | | | | | |
| Diabetes | O | Binary variable equal to one if diagnosed with diabetes. Only available for individuals who have died. | | | | | | |

**Table S2.** Descriptive statistics for categorical and binary variables at individual level. Share (%) belonging to each group/category.

|  | All | Dead | Dead in COVID-19 |
| --- | --- | --- | --- |
| Observations | 8,477,638 | 97,197 | 10,154 |
| *Ethnic background* |  |  |  |
| Sweden | 78.3 | 86.2 | 79.6 |
| Nordic | 2.62 | 5.56 | 6.85 |
| EU15 | 1.78 | 1.76 | 2.09 |
| West Balkan | 1.75 | 1.38 | 1.71 |
| East Europe | 3.06 | 1.78 | 2.15 |
| Middle East | 4.70 | 1.49 | 4.00 |
| East Africa | 1.61 | 0.31 | 0.99 |
| North-South-West Africa | 0.79 | 0.17 | 0.31 |
| South-Central Asia | 2.47 | 0.54 | 1.06 |
| Southeast-East Asia | 1.57 | 0.27 | 0.39 |
| South-Central America | 1.01 | 0.37 | 0.68 |
| North America-Oceania | 0.34 | 0.16 | 0.15 |
| *Education* |  |  |  |
| Elementary school | 19.1 | 42.8 | 42.3 |
| High school | 41.4 | 37.8 | 36.8 |
| Shorter higher education | 13.8 | 7.63 | 7.62 |
| Longer higher education | 21.8 | 9.77 | 9.73 |
| Unknown | 3.96 | 2.07 | 3.57 |
| *Age* |  |  |  |
| 15-29 | 22.3 | 0.74 | 0.14 |
| 30-39 | 16.1 | 0.77 | 0.27 |
| 40-49 | 15.3 | 1.43 | 0.69 |
| 50-59 | 15.3 | 3.96 | 2.39 |
| 60-69 | 13.1 | 9.87 | 6.40 |
| 70-79 | 11.7 | 24.4 | 22.1 |
| 80-89 | 5.15 | 35.9 | 42.7 |
| 90+ | 1.17 | 22.9 | 25.4 |
|  |  |  |  |
| Female | 50.0 | 49.7 | 46.7 |
|  |  |  |  |
| *Civil status* |  |  |  |
| Single | 40.6 | 67.3 | 69.4 |
| Single with child | 5.21 | 0.32 | 0.10 |
| Married | 28.1 | 31.1 | 29.5 |
| Married with child | 26.1 | 1.23 | 0.97 |
|  |  |  |  |
| Frontline worker | 10.1 | 1.68 | 1.08 |
|  |  |  |  |
| Elderly home | 1.00 | 18.1 | 24.0 |
|  |  |  |  |
| *House type* |  |  |  |
| Rental (apartment) | 30.2 | 44.0 | 49.3 |
| Tenant-owned (apartment) | 20.4 | 18.8 | 20.9 |
| Owner-occupied (house) | 47.2 | 31.8 | 22.4 |
| Unknown | 2.18 | 5.40 | 7.37 |
|  |  |  |  |
| Inter-generational household | 0.86 | 1.19 | 1.57 |
|  |  |  |  |
| Crowded household | 9.24 | 2.31 | 2.96 |
|  |  |  |  |
| *Multiple causes of death* |  |  |  |
| One cause | - | 27.2 | 4.77 |
| Two causes | - | 27.3 | 19.2 |
| Three causes | - | 22.2 | 28.5 |
| Four causes | - | 13.3 | 25.0 |
| Five or more causes | - | 9.96 | 22.5 |
|  |  |  |  |
| Diabetes | - | 12.2 | 18.4 |

**Table S3.** Descriptive statistics for continuous variables.

| Variable | Population | Observations | Mean | Std. Dev. | Min | Max |
| --- | --- | --- | --- | --- | --- | --- |
| Age | All | 8,477,503 | 48.10 | 19.93 | 15 | 111 |
|  | Dead | 97,197 | 79.86 | 12.90 | 16 | 111 |
|  | Dead in COVID-19 | 10,154 | 82.45 | 10.38 | 18 | 107 |
| Income (ln) | All | 8,477,638 | 7.707 | 1.127 | 0 | 17.59 |
|  | Dead | 97,197 | 7.563 | 0.7073 | 0 | 13.46 |
|  | Dead in COVID-19 | 10,154 | 7.587 | 0.6374 | 0 | 12.31 |
| Virus exposure (ln) | Dead | 97,197 | 4.752 | 3.0205 | 0 | 10.65 |
|  | Dead in COVID-19 | 10,154 | 6.521 | 1.942 | 0 | 10.65 |
| Population density (ln) | All | 8,477,638 | 4.950 | 1.997 | -1.609 | 8.705 |
|  | Dead | 97,197 | 4.624 | 1.992 | -1.609 | 8.705 |
|  | Dead in COVID-19 | 10,154 | 5.361 | 2.107 | -1.204 | 8.705 |

**Table S4.** Descriptive statistics for neighborhood variables.

|  | **Ethnic** | **Low education** | **Poverty** |
| --- | --- | --- | --- |
| Observations | 8,477,638 | 8,477,638 | 8,477,638 |
| Mean | 0.1729 | 0.1155 | 0.1632 |
| Standard deviation | 0.1540 | 0.0645 | 0.0940 |
| Minimum | 0.0112 | 0.0010 | 0.0148 |
| Maximum | 0.8166 | 0.4624 | 0.6944 |
| *Percentiles* |  |  |  |
| 1% | 0.0211 | 0.0243 | 0.0430 |
| 5% | 0.0322 | 0.0371 | 0.0599 |
| 10% | 0.0428 | 0.0467 | 0.0717 |
| 25% | 0.0713 | 0.0690 | 0.1003 |
| 50% (median) | 0.1235 | 0.1032 | 0.1402 |
| 75% | 0.2120 | 0.1466 | 0.1992 |
| 90% | 0.3833 | 0.2008 | 0.2805 |
| 95% | 0.5346 | 0.2387 | 0.3527 |
| 99% | 0.7202 | 0.3310 | 0.5135 |

**Table S5.** Countries included in each region of origin.

| **Region of origin** | **Countries** |
| --- | --- |
| Sweden | Sweden |
| Nordic | Denmark, Finland, Norway, Iceland |
| EU15 | Austria, Belgium, France, Germany, Greece, Ireland, Italy, Luxembourg, Netherlands, Portugal, Spain, United Kingdom |
| West Balkan | Albania, Bosnia-Hercegovina, Kosovo, Macedonia, Montenegro, Yugoslavia |
| East Europe | Andorra, Bulgaria, Croatia, Cyprus, Czech Republic, Estonia, Hungary, Latvia, Liechtenstein, Lithuania, Malta, Moldavia, Monaco, Poland, Romania, Russia, San Marino, Serbia, Slovakia, Slovenia, Switzerland, Vatican City State |
| Middle East | Armenia, Azerbaijan, Bahrain, Georgia, ‎Iraq, Israel, Jordan, Kuwait, Lebanon,‎ Oman, Palestine, Qatar‎, Saudi Arabia, Syria, Turkey, United Arab Emirates, Yemen |
| East Africa | Burundi, Comoros, Djibouti, Eritrea, Ethiopia, Kenya, Madagascar, Malawi, Mauritius, Mozambique, Rwanda, Seychelles, Somalia, Tanzania, Uganda, Zambia, Zanzibar, Zimbabwe |
| North-South-West Africa | All other countries in Africa |
| South-Central Asia | Afghanistan, Bangladesh, Bhutan, India, Iran, Kazakhstan, Kyrgyzstan, Maldives, Nepal, Pakistan, Sikkim, Sri Lanka, Tajikistan, Turkmenistan, Uzbekistan |
| Southeast-East Asia | Brunei, Burma (Myanmar), Cambodia, China, Hong Kong, Indonesia, Japan, Laos, Malaysia, Mongolia, North Korea, the Philippines, Singapore, South Korea, Taiwan, Thailand, Timor-Leste, Vietnam |
| South-Central America | All countries in South and Central America |
| North America-Oceania | All countries in North America and Oceania |

**Table S6**. Marginal effects for the probability of dying with COVID-19 at the individual level, probit estimations without selection bias correction.

|  | (1b’) | | (2b’) | | (3b’) | |
| --- | --- | --- | --- | --- | --- | --- |
| *Neighborhood characteristics* | i) Ethnic | | ii) Low education | | iii) Poverty | |
|  | >7-15%  >15-25%  >25-35%  >35-45%  >45-55%  >55-65%  >65-75%  >75% | .0075*  .0147*  .0134*  .0310*  .0265*  .0359 *  .0530*  .0933* | >7-10%  >10-15%  >15-20%  >20-25%  >25-30%  >30-35%  >35% | -.0013  -.0018  .0036  .0129*  .0338*  .0347*  .0708* | >10-17.5%  >17.5-25%  >25-32.5%  >32.5-40%  >40-47.5%  >47.5-55%  >55% | .0008  -.0005  .0101*  .0266*  .0454*  .0158  .0799* |
| No-go-zone | -.0043 | | -.0011 | | .0022 | |
| *Regional characteristics* | | | | | | |
| Virus exposure | .0215* | | .0216* | | .0216* | |
| Population density | -.0078* | | -.0065* | | -.0071* | |
| Stockholm | .0619* | | .0634* | | .0654* | |
| *Individual characteristics* | | | | | | |
| Ethnic background  Nordic  EU15  West Balkan  East Europe  Middle East  East Africa  North-South-West Africa  South-Central Asia  Southeast-East Asia  South-Central America  North America-Oceania | .0128*  .0058  .0230*  .0096  .1121*  .2216*  .0487  .0797*  .0586*  .0532*  -.0127 | | .0134*  .0064  .0261*  .0111  .1204*  .2206*  .0510  .0856*  .0615*  .0561*  -.0118 | | .0136*  .0064  .0250*  .0108  .1184*  .2247*  .0524  .0847*  .0621*  .0565*  -.0126 | |
| Education  High school  Shorter higher education  Longer higher education | .0011  .0001  -.0066 | | .0012  -.0000  -.0072 | | .0010  -.0004  -.0074 | |
| Income | .0036 | | 0.0036 | | 0.0037 | |
| Age  30 – 39  40 – 49  50 – 59  60 – 69  70 – 79  80 – 89  90 + | .0186  .0344*  .0453*  .0516*  .0745*  .0951*  .0922* | | .0188  .0342*  .0451*  .0515*  .0744*  .0951*  .0917* | | .0189  .0340*  .0453*  .0516*  .0746*  .0952*  .0920* | |
| Female | -.0165* | | -.0165* | | -.0164* | |
| Civil status  Single with child  Married  Married with child | -.0317  .0013  .0277 | | -.0307  .0013  .0265 | | -.0319  .0014  .0272 | |
| Frontline worker | .0089 | | .0099 | | .0099* | |
| Elderly home | .0300* | | .0296* | | .0295* | |
| House type  Tenant-owned (apartment)  Owner-occupied (house) | .0024  -.0031 | | .0027  -.0057 | | .0024  -.0059 | |
| Inter-generational household | .0091 | | .0092 | | .0091 | |
| Crowded household | .0002 | | .0000 | | -.0002 | |
| Multiple causes of death  Two causes  Three causes  Four causes  Five or more causes | .0567*  .1109*  .1676*  .2057* | | .0568*  .1109*  .1680*  .2060* | | .0568*  .1110*  .1679*  .2060* | |
| Diabetes | -.0149* | | -.0150* | | -.0149* | |
| Observations | 97,192 | | 97,192 | | 97,192 | |
| Pseudo R2 | .1912 | | .1909 | | .1909 | |
| Wald Chi2 | 9,818* | | 9,836* | | 9,817* | |

*Notes:* * denote significance at 1% level. Robust standard errors in parenthesis.

1. We have also tested two weeks before, with robust results. [↑](#footnote-ref-1)
2. This variable is not available at the neighborhood level. [↑](#footnote-ref-2)
3. North America and Oceania are grouped together due to the relatively low number of foreign-born individuals with origin in these regions, as well as the sharing of similar cultural backgrounds. [↑](#footnote-ref-3)
4. In Sweden, households are classified as crowded if there are less rooms than household members, with the exception that married/co-habiting adults are assumed to share a bedroom. The number of rooms do not include a kitchen and a living room. Hence, single individuals living in one-room apartments are classified as crowded according to Swedish standards. [↑](#footnote-ref-4)
